# Whole-Slide Image Analysis of Human Pancreas Samples to Elucidate the Immunopathogenesis of Type 1 Diabetes Using the QuPath Software

**DOI:** 10.3389/fmolb.2021.689799

**Published:** 2021-06-11

**Authors:** Paola S. Apaolaza, Peristera-Ioanna Petropoulou, Teresa Rodriguez-Calvo

**Affiliations:** ^1^ Institute of Diabetes Research, Helmholtz Diabetes Center at Helmholtz Zentrum München, Munich, Germany; ^2^ German Center for Diabetes Research (DZD), Helmholtz Zentrum Munich, Munich, Germany

**Keywords:** pancreas, type 1 diabetes, pathology, multiplex immunofluorescence, whole-slide image analysis, QuPath

## Abstract

Type 1 diabetes is a chronic disease of the pancreas characterized by the loss of insulin-producing beta cells. Access to human pancreas samples for research purposes has been historically limited, restricting pathological analyses to animal models. However, intrinsic differences between animals and humans have made clinical translation very challenging. Recently, human pancreas samples have become available through several biobanks worldwide, and this has opened numerous opportunities for scientific discovery. In addition, the use of new imaging technologies has unraveled many mysteries of the human pancreas not merely in the presence of disease, but also in physiological conditions. Nowadays, multiplex immunofluorescence protocols as well as sophisticated image analysis tools can be employed. Here, we described the use of QuPath—an open-source platform for image analysis—for the investigation of human pancreas samples. We demonstrate that QuPath can be adequately used to analyze whole-slide images with the aim of identifying the islets of Langerhans and define their cellular composition as well as other basic morphological characteristics. In addition, we show that QuPath can identify immune cell populations in the exocrine tissue and islets of Langerhans, accurately localizing and quantifying immune infiltrates in the pancreas. Therefore, we present a tool and analysis pipeline that allows for the accurate characterization of the human pancreas, enabling the study of the anatomical and physiological changes underlying pancreatic diseases such as type 1 diabetes. The standardization and implementation of these analysis tools is of critical importance to understand disease pathogenesis, and may be informative for the design of new therapies aimed at preserving beta cell function and halting the inflammation caused by the immune attack.

## Introduction

The pancreas is mainly divided into exocrine and endocrine tissue. The islets of Langerhans, which account for 1–4% of the total pancreatic volume, form the endocrine portion and contain several cell populations (secreting distinct proteins): alpha cells (glucagon), beta cells (insulin), delta cells (somatostatin), epsilon cells (ghrelin), and pancreatic polypeptide cells (PP) (Dolenšek et al., 2015; Noguchi and Huising, 2019). In physiological conditions, beta cells constitute approximately 50–70% of the total endocrine cells, followed by alpha cells (20–40%), delta cells (<10%), and a few epsilon and PP cells (Steiner et al., 2010); however these proportions vary from region to region (e.g., PP cells can reach 80% in the pancreatic head, whereas beta cells are <20% in the same region) or with disease stage (Rahier et al., 1983; Steiner et al., 2010). The exocrine pancreas accounts for 96–99% of the total volume and is organized into lobes, lobules, and acini (dome-like structures consisting of acinar cells); single endocrine cells can be found throughout the acinar and ductal tissue (Dolenšek et al., 2015). Both endocrine and exocrine tissue are affected in type 1 diabetes (T1D) (Rodriguez-Calvo et al., 2014; Campbell-Thompson et al., 2015; Alexandre-Heymann et al., 2019; Bender et al., 2020), but the disease is characterized by a chronic autoimmune destruction of insulin-producing beta cells (Rowe et al., 2011). Recently, in individuals with recent onset T1D, a decrease in pancreatic volume has been observed compared to healthy controls (Williams et al., 2012), indicating that pancreatic atrophy might be an important contributing factor to disease pathogenesis and bringing the often-neglected study of the pancreas as a whole to T1D research.

Beta cells are the main source of insulin biosynthesis, storage, and secretion (Vasiljević et al., 2020). Insulin is a peptide hormone of 51 amino acids consisting of two chains (A and B chain, linked by two disulfide bonds), which is initially synthesized from a single-chain precursor—preproinsulin. After synthesis in the ribosomes, preproinsulin is transferred to the endoplasmic reticulum (ER), where proinsulin is created by cleavage of the signal peptide. When folding and disulfide bonds are completed, proinsulin is transferred to the Golgi, where it is packaged in clathrin-coated vesicles (Steiner et al., 2009; Vasiljević et al., 2020). In these granules, proinsulin is cleaved sequentially by 1) prohormone convertase 1/3 (PC1/3), which shows preference for the C-peptide/A-chain junction, but cleaves also at the C-peptide/B-chain junction, 2) prohormone convertase 2 (PC2), which cleaves at the C-peptide/B-chain junction, and 3) carboxypeptidase E (CPE), which cleaves away the connecting segment and removes any remaining C-terminal basic residues from both insulin and C-peptide (Steiner et al., 2009; Vasiljević et al., 2020). In T1D and other pancreatic diseases, alterations at the level of these enzymes in beta cells have a major impact in proinsulin processing, proinsulin and insulin secretion (Sims et al., 2019), and overall beta cell function.

Infiltrating immune cells can be found scattered in the exocrine and endocrine pancreas in physiological conditions and their numbers increase prior to, at the time of diagnosis, and after T1D onset (Willcox et al., 2009; Rodriguez-Calvo et al., 2014; Bender et al., 2020). Insulitis, which is a hallmark of T1D, has been defined over the years as infiltration by ≥15 CD45+ cells (Campbell-Thompson et al., 2013) or ≥6 CD3+ cells (Campbell-Thompson et al., 2016) located immediately adjacent to or within the islet, in a minimum of three islets of standard size (150 μm of diameter). In addition, pseudoatrophic islets (insulin deficient) should be present in the tissue section (Campbell-Thompson et al., 2013). T cells are the major cell type found in insulitis, and their presence is significantly higher in T1D subjects, not only in the islets, but also in the exocrine compartment (Rodriguez-Calvo et al., 2014). Up to this date, the events leading to the autoimmune attack and the consequent beta cell destruction are not well elucidated. However, increasing evidence suggests a potential self-involvement of beta cells in their own demise (Mallone and Eizirik, 2020; Roep et al., 2020). In individuals with genetic predisposition, intrinsic properties of beta cells such as high ER stress, vascularization, and hormone secretion, might intensify the presentation of self-antigens on beta cells, which could increase the recruitment of immune cells to the islets.

Early research in T1D was originally based on limited human pancreatic specimens (Foulis and Stewart, 1984; Foulis et al., 1986), experimental mouse models [mainly the non-obese diabetic (NOD) mouse (Anderson and Bluestone, 2005)], or on beta cell lines (Scharfmann et al., 2019). During the last decades, the scientific community realized the importance of systematic organ collection and distribution for research and founded several biobanks, such as the Exeter Archival Diabetes Biobank (EADB) (Foulis et al., 1986), the Dutch Pancreas Biobank (Strijker et al., 2018), and the IMIDIA Biobank (Solimena et al., 2018). The National Institutes of Health established the Human Pancreas Analysis Program (HPAP), which aims to distribute high quality molecular data derived from human pancreata in order to enable scientific discovery (Kaestner et al., 2019). The biggest and well-known biobank in the T1D field is the Network for Pancreatic Organ Donors with Diabetes (nPOD), founded by the Juvenile Diabetes Research Foundation (JDRF) in 2007, and based in the United States (Campbell-Thompson et al., 2012). nPOD’s main goals are to obtain and distribute pancreatic or disease-relevant tissue samples from organ donors to affiliated researchers around the globe, as well as to foster and promote collaboration between research teams, leading ultimately to a quicker elucidation of the disease pathogenesis (Pugliese et al., 2014).

One of the biggest challenges in the study and analysis of pancreas pathology is the heterogeneity of the human pancreas, which is evident at the beta cell, the islet, and the organ level (Dybala and Hara, 2019). Manual analysis of multiple regions of interest (ROIs) has been traditionally performed, which faced a lack of robustness and reproducibility. This type of analysis is prone to bias and cannot capture the variability in size, endocrine composition, architecture, vasculature and immune cell infiltration in islets, and exocrine tissue (Dybala and Hara, 2019). Several algorithms and workflows for the analysis of 2D (Wang et al., 2013; Kilimnik et al., 2012) and 3D (Poudel et al., 2016; Fowler et al., 2018) images of the pancreas have been proposed using specific Fiji plugins and MATLAB. However, most of these algorithms are not intuitive and require a considerable amount of computer proficiency. Nowadays, whole-slide image analysis of tissue sections, provided by the biobanks mentioned above, is becoming increasingly accessible to researchers. To date, analysis of such images required either specialized commercial software (Tang et al., 2020) or was limited to a small ROI, due to the inability of existing open-source software to handle large 2D images (Aeffner et al., 2019). QuPath (http://qupath.github.io) is an open-source, user-friendly software developed by Bankhead et al. (2017) in 2016 in order to enable whole-slide image analysis and digital pathology, by addressing the unique requirements in the visualization and analysis of such data.

Here, we show that QuPath can automatically and accurately detect, quantify and distinguish cell populations in the endocrine and the exocrine compartments of the pancreas using a series of detection algorithms based on intensity thresholding, pixel classification, and machine learning. We provide the groundwork for a standardized, semi-automated analysis of the human pancreas using QuPath, which can lead to a more efficient and reproducible analysis of tissue images, reducing inter-observer variability, and bringing researchers closer to elucidating the etiology of T1D.

## Materials and Equipment

### QuPath Software

Multiplexed fluorescence images from tissue sections were analyzed with QuPath version 0.2.3, an open-source software for digital pathology and whole-slide image analysis described by Bankhead et al. (2017). Briefly, the software was developed using Java 8, with a JavaFX interface for annotation and visualization, built-in algorithms for common tasks, including cell and tissue detection, and interactive machine learning for object and pixel classification. It is compatible with ImageJ, OpenCV, Java Topology Suite, and OMERO. The software supports several image formats through Bio-Formats and OpenSlide, including whole-slide images and multiplexed data.

### Pancreatic Specimens

Six 4-µm-thick pancreatic formalin-fixed paraffin-embedded (FFPE) sections from the tail of the pancreas of a female non-diabetic donor, were obtained through nPOD. All the sections were obtained from the same tissue block. Slides #1, #2, #3, #4, and #6 were consecutive, while section #5 was not. Briefly, the donor was 64 years old, Caucasian, with a BMI of 31.2 and an HLA-A*02/03, B*07/60, DR*13/15, DQ*06 phenotype, who was hospitalized for 2.67 days due to a cerebrovascular accident. The histopathology record showed insulin and glucagon positive normal islets. All experimental procedures were approved by the ethics committee at the Technical University of Munich (protocol #215/17 S) and the Helmholtz Center Munich, Institute of Diabetes Research.

### Immunofluorescence and Imaging

FFPE sections were stained for insulin, proinsulin, glucagon, CD3, CD8, CD45, chromogranin A (CHGA), PC1/3, PC2, and CPE by immunofluorescence (Supplementary Figure S1). Tissue sections were deparaffinized with an alternative to xylene clearing agent (H2779, Sigma-Aldrich, MO, United States) and rehydrated in ethanol baths of decreasing ethanol content. Antigen retrieval and multiplexing of primary antibodies of the same species was performed using the Opal kit according to the manufacturer’s instructions (NEL811001KT, Akoya Biosciences, CA, United States). Specifically, a 2-step microwave antigen retrieval process preceded the primary antibody incubations and was the same for all the stainings. Slides were first microwaved at 900 W for 45–65 s (until retrieval buffer reached the boiling point), followed by a second step, where the sections were microwaved for 15 min at 160 W. The following primary antibodies were incubated for 1 h at room temperature or overnight at 4°C depending on the protocol: mouse anti-proinsulin (1:200, GS-9A8 supernatant, DSHB, IA, United States), mouse anti-CD45 (1:100, M070101, Agilent Technologies, CA, United States), rabbit anti-CD3 (1:200, A045229, Agilent Technologies), rabbit anti-CD8 (1:900, ATA-HPA037756, Atlas Antibodies, Bromma, Sweden), mouse anti-insulin (1:300, 5-1108, Merck, Darmstadt, Germany), guinea pig anti-insulin (1:500; A056401-2, Agilent Technologies), rabbit anti-glucagon (1:1200; ab92517, Abcam, Cambridge, United Kingdom), rabbit anti-CHGA (1:500; ab15160, Abcam), mouse anti-PCSK1N (1:500, ATA-HPA064734, Atlas Antibodies), rabbit anti-PC2 (1:800, Merck), and rabbit anti-CPE (1:100, ATA-HPA003819, Atlas Antibodies). Detection was performed by 1 h incubation at room temperature with the following secondary antibodies at 1:1,000 dilution (all from Life technologies, Darmstadt, Germany): Goat Anti-Guinea Pig IgG Alexa Fluor 488 (A11073), Goat Anti-Rabbit IgG Alexa Fluor 750 (A21039), Goat Anti-Mouse IgG Alexa Fluor 750 (A21037), F(ab’)2-Goat anti-Rabbit IgG Alexa Fluor 488 (A11070), Goat Anti-Mouse IgG1 Alexa Fluor 555 (A21127), F(ab’)2-Goat anti-Rabbit IgG Alexa Fluor 555 (A21430), and Goat Anti-Mouse IgG1 Alexa Fluor 647 (A21240). Sections were counterstained with Hoechst 33342 (1:5,000; Invitrogen, CA, United States) and mounted with Prolong Gold Antifade reagent (Invitrogen). Whole tissue sections were scanned by an Axio Scan.Z1 slide scanner (Zeiss, Jena, Germany) using a 20x/0.8NA Plan-Apochromat (a = 0.55 mm) objective.

### Statistics

All the graphs show the median and 95% confidence interval of the median. Analyses were performed using GraphPad Prism version 9, GraphPad Software, La Jolla, CA, United States, www.graphpad.com.

### Standard Operating Procedure for Whole Slide Image Analysis

A step by step guide and detailed information on how to analyze whole-slide pancreatic tissue sections is provided as a supplementary document (Supplementary Data S1).

## Methods

### Tissue, Islet and Cell Detection

**FIGURE 1:**
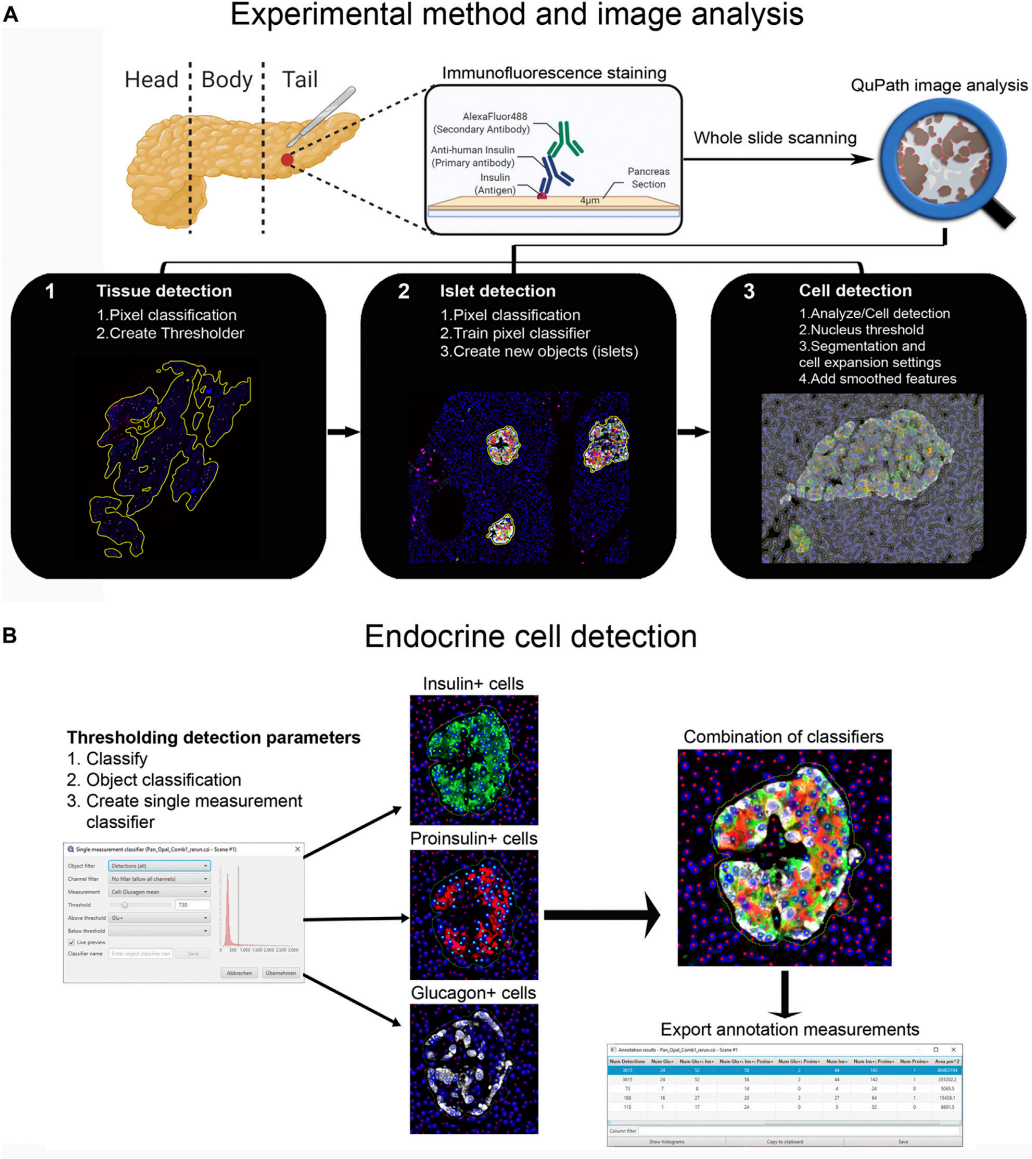
Schematic illustration of the whole-slide image analysis workflow using QuPath. **(A)** Experimental method and image analysis. Multiple immunofluorescence protocols were employed for the staining of tissue sections from the pancreatic tail of a single non-diabetic donor. Whole-slide image analysis was carried out with QuPath, version 0.2.3 1); tissue was detected using an intensity thresholder based on average values of all channels for the labeled proteins 2), Objects (islets) were then created using the pixel classifier, and 3) cells were detected and smoothed features were added. **(B)** The *Single measurement classifier tool* was employed to detect positive cells for the marker of interest. Cells were identified as areas of staining above the background level by applying optimized *Cell mean* intensity thresholds. Combination of single classifiers was necessary for the accurate detection of beta and alpha cells. Annotation measurements were exported as CSV files and were subsequently processed in Excel spreadsheets.

First, tissue area and islets were automatically identified based on average values of all channels for the labeled proteins (antibody combinations are shown in Supplementary Table S1) using thresholding detection and machine learning. Information on the whole tissue section, exocrine and endocrine areas, number of islets per section, as well as the total number of alpha and beta cells was obtained. Independent workflows and settings for 1) tissue, 2) islet, and 3) cell detection are shown in Figure 1A and Supplementary Figure S2. Only islets formed by ≥10 cells were included in the analysis in order to avoid scattered single endocrine cells present in the exocrine tissue and possible detection errors derived from small artefacts. First, for tissue detection, the command *Pixel classification → Create thresholder* was used (Figure 1A). After applying the *fill holes* function, the tissue was manually checked for the presence of artifacts. Then, a small ROI was created, and islets were recognized as a new class by *Pixel classification*. For this purpose, the command *Train pixel classifier* was used and new objects (islets) were created. Once the new islet classifier was saved, *Cell detection* was performed in the entire tissue section. Cells were identified as areas of staining above the background level, by applying optimized *nucleus threshold*, segmentation parameters (*Median filter radius and Sigma*), and cell expansion (Figure 1A and Supplementary Figure S2). Last, *smoothed features* were added in order to obtain new measurements considering the cell features within a 25 µm range. After cells were detected, the islet pixel classifier, initially applied to a small ROI, was applied to the whole tissue area, and the newly created islet areas, defined as objects, were filled automatically following the path *Objects→ Annotations→ Fill holes*.

### Endocrine Cell Detection

Thresholding detection was applied to create unique classifiers for every staining combination due to fluorescence channel dependency. After islet detection, the path *Classify* → *Object classification* → *Create single measurement classifier tool* was applied to detect cells positive for insulin, proinsulin, glucagon, PC1/3, PC2 or CPE (Figure 1B). Cells were identified as areas of staining above the background level by applying optimized *Cell mean* intensity thresholds. To identify beta cells, the new classifiers were combined to obtain the number of cells positive for both insulin and proinsulin together with different proteins of interest like PC1/3, PC2 or CPE. Data on alpha cells were obtained by using glucagon positive cells as reference. Chromogranin A was used for complete islet cell detection for slide #6. *Annotation measurements* were exported and information on islet size, cell composition and number of positive cells was obtained (Table 1 and Supplementary Table S2).

**TABLE 1:** Characterization of the endocrine and the exocrine pancreas of a non-diabetic donor according to different staining combinations.

Slide ID	Tissue area (mm^2^)	Exocrine area (mm^2^)	Endocrine area (mm^2^)	No. islets	No. islet cells	No. beta cells	No. alpha cells
1-PI/CD45/INS/GCG	80.9	79.8	1.2	260	13,625	9,571	4,537
2-INS/PI/GCG	71.8	70.6	1.3	262	13,129	7,581	4,163
3-INS/PC1/PI	84.6	83.4	1.1	234	12,441	9,040	3,401
4-INS/CPE/PI	92.9	91.7	1.2	273	14,616	11,682	2,934
5-INS/PC2/PI	130.6	128.3	2.3	432	26,655	17,579	9,076
6-CD3/CD8/CHGA	75.0	74.14	0.9	241	13,203	NA	NA
Total mean ± SD	89.3 ± 19.7	87.9 ± 19.2	1.3 ± 0.5	283.7 ± 67.6	15,611.5 ± 4,981.8	11,090.6 ± 3,500.9	4,822.2 ± 2,199.8

### Immune Cell Detection and Spatial Analysis of Immune Infiltration

**FIGURE 2:**
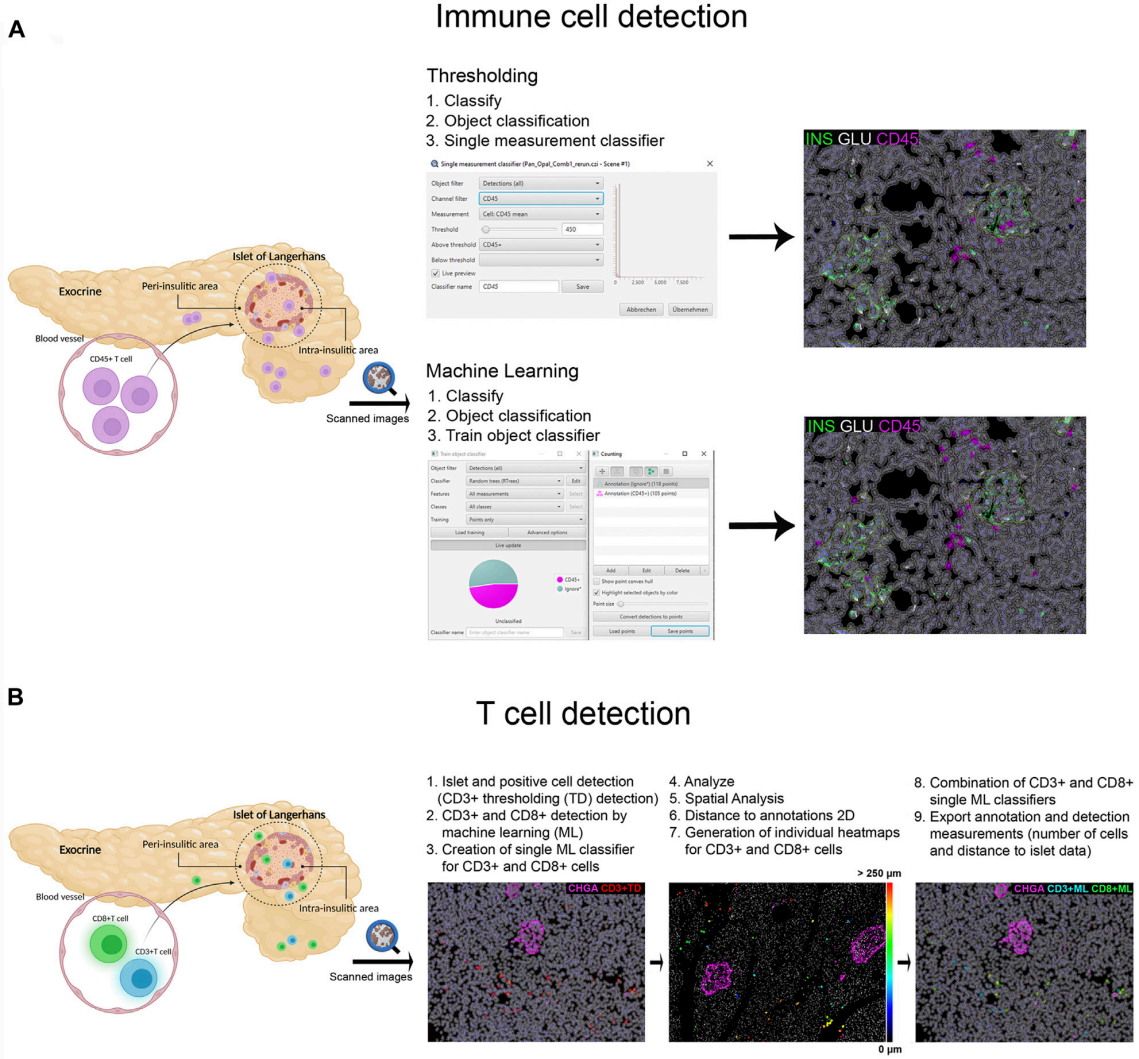
Schematic illustration of the immune cell detection workflow using QuPath. **(A)** Comparison of the thresholding (single measurement classifier) and the machine learning (train object classifier) option for the detection of CD45+ cells. **(B)** Workflow for the detection and distance analysis of CD3+ and CD8+ cells detected with thresholding and machine learning. Heatmaps were created to depict the relative distance of CD3+ and CD8+ cells (either individually or combined as one class) to the annotated islets.

Different image analysis protocols were generated for the study of CD45+ (leucocyte marker), CD3+ (T cell marker) and CD8+ cells (CD8+ T cell marker) and their localization in the islets and exocrine tissue. CD4+ T cells were calculated as the total number of CD3+ cells minus the number of CD8+ cells (CD3+ CD8−cells). The use of thresholding vs. machine learning was compared (Figure 2A). First, the membrane marker CD45, which is expressed in all leucocytes, was detected. For thresholding, a *single measurement classifier* for the *cell mean* intensity value of the CD45 marker was used. Using this method, an overestimation in the number of islet-infiltrating cells was observed, and manual correction was applied. For machine learning, the following path was used: *Classify → Object classification → Train object classifier*. For the classifier training, the option *Points only* was selected. Then, the *Points* tool was used to assign two different classes to the corresponding cells, one for the marker of interest (CD45+), and one for unclassified objects (ignore*)*.* For each class, negative (ignore*) and positive (CD45+), three different ranges of training points were tested (≥50, ≥80, and over 100). Overall, classifying between 50 and 80 points and using machine learning was comparable to applying the best threshold and subsequent manual correction. Moreover, when the whole section was analyzed, the total number of CD45+ cells detected by thresholding was lower than the one obtained by machine learning using 100 training points (8,078 vs. 17,116 cells), indicating that immune cells with low intensity values were not properly detected when thresholding was used (Table 2). Machine learning using ≥100 points showed higher accuracy than thresholding and was subsequently used for the detection of CD3+ and CD8+ cells. However, the most suitable number of training points should be defined by the user based on staining, intrinsic characteristics of the tissue and quality of the specimen. Last, measurements were exported and the number, proportion of infiltrated islets and density of CD45+ cells (expressed as number of positive cells per mm^2^) were calculated in the whole tissue, and the exocrine and endocrine compartments (Table 3 and Figure 6).

**TABLE 2:** Comparison of the number of CD45+ cells in the whole tissue, the exocrine and endocrine area of slide #1, depending on the detection method (thresholding vs. machine learning).

QuPath method	Total no. CD45+	No. CD45+ in exocrine	No. CD45+ in endocrine
Thresholding	8,107	8,069	38
Thresholding manually corrected	8,078	8,067	11
Machine learning (≥50 points)	6,801	6,800	1
Machine learning (≥80 points)	12,874	12,870	4
Machine learning (≥100 points)	17,116	17,098	18

**TABLE 3:** Number, proportion and density of immune cells in the whole tissue, the exocrine and endocrine area. CD45+ cell values correspond to the analysis of slide #1, and CD3+, CD8+, and CD4+ cell analysis to slide #6.

	No. CD45+	No. CD3+	No. CD8+	No. CD4+	% CD45+	% CD3+	% CD8+	% CD4+	CD45+/mm^2^	CD3+/mm^2^	CD8+/mm^2^	CD4+/mm^2^
Tissue	17,116	8,643	3,911	4,732	100	100	100	100	211.5	115.2	52.1	63.1
Exocrine	17,098	8,639	3,909	4,730	99.9	99.95	99.95	99.96	214.3	116.7	52.8	63.9
Endocrine	18	4	2	2	0.1	0.05	0.05	0.04	15.5	4.1	2	2

In order to characterize T cell infiltration, a modified version for cell detection was applied as follows (Figure 2B): After islets were detected, the option *Positive cell detection* was used to identify by thresholding all CD3+ cells. However, as cell detection by thresholding was not completely accurate, a second classifier for CD3+ or CD8+ membrane markers was created using machine learning. As explained above, the object classifier was trained with a minimum of 100 training points for CD3+ and CD8+ cell detection, and was applied over the CD3+ cells detected by thresholding, creating a single machine learning classifier for CD3+ and CD8+ cells. Then, once T cells were identified, their localization with respect to the islets was analyzed (Figure 2B). Distance analysis was performed using the command *Spatial analysis*. This tool was applied as follows: *Analyze→ Spatial analysis→ Distance to annotations 2D*. Next, we generated individual heatmaps for CD3+ and CD8+ cells based on the distance of the cells to the islets. Finally, both single machine learning classifiers were combined and different classes were automatically created; the first one corresponding to all CD3+ cells, the second class for cells positive for both markers (CD3+CD8+), representing CD8+ cells, and the third one representing CD4+ cells (CD3+CD8−). Last, *annotation* and *detection measurements* were exported. Data on T cell numbers, endocrine and exocrine T cell density, proportion of infiltrated islets, as well as the distance of T cells to the islets were obtained (Table 3 and Figures 6, 7).

## Results

### Characterization of the Endocrine and Exocrine Pancreas in a Non-diabetic Donor

**FIGURE 3:**
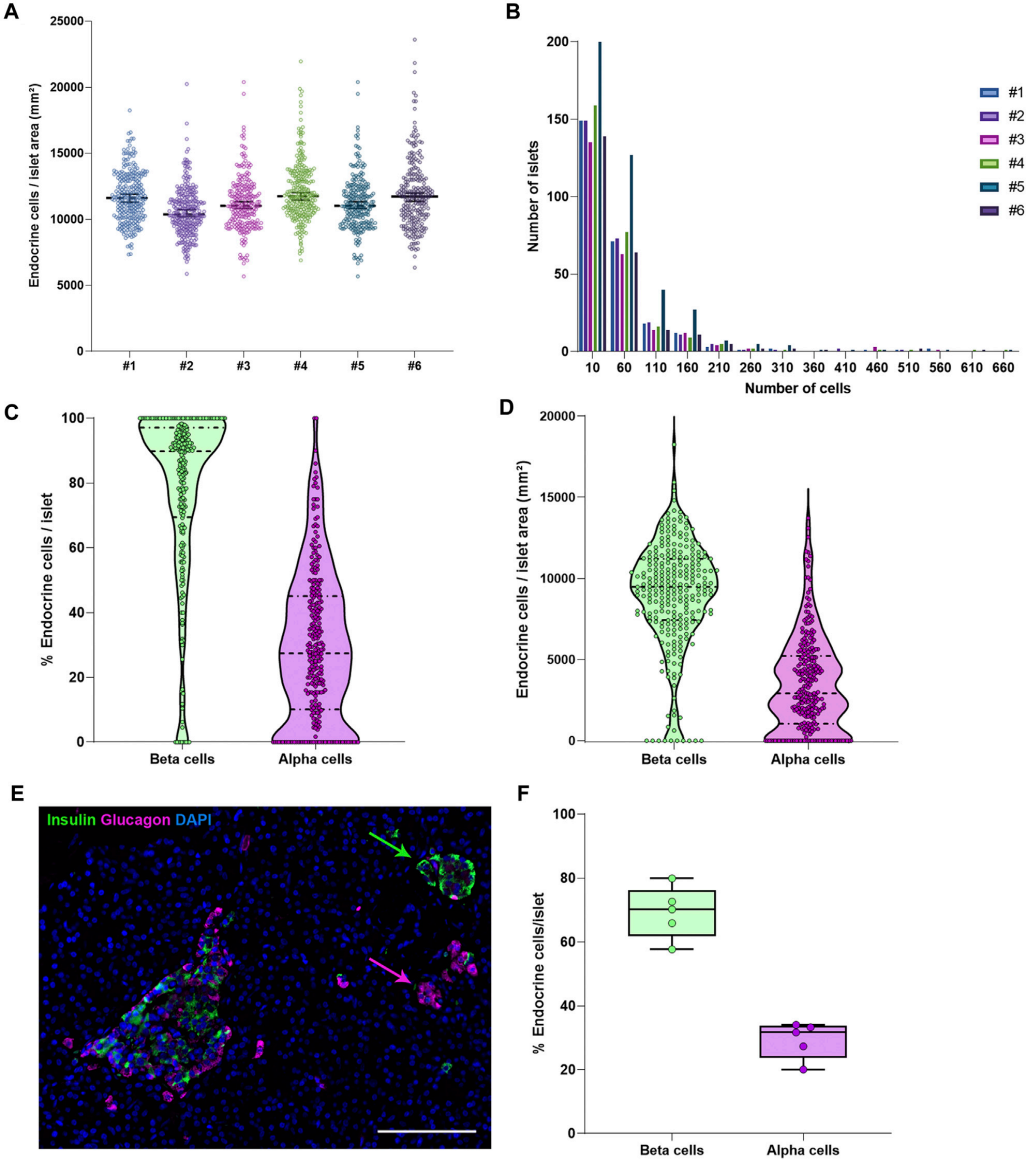
Characterization of the endocrine and exocrine pancreas in a non-diabetic donor. **(A)** Comparison of islet density expressed as number of endocrine cells per islet area in mm^2^ among the different staining combinations. Each dot represents an islet. **(B)** Histograms showing the cellular content of islets in pancreatic sections stained with different antibody combinations. The different staining IDs are shown as #1–6. Each bar represents a different slide. More details can be found in Supplementary Table S1. **(C)** Violin plots showing the percentage of endocrine cells (beta and alpha cells) per islet analyzed in the whole pancreatic section, stained with antibody combination #1. Each dot colored represents an islet. **(D)** Violin plots showing the density of beta and alpha cells in the same section, expressed as number of endocrine cells per mm^2^ of islet area. Each dot represents an islet. **(E)** Representative image showing two islets, one containing mainly beta cells (green) and one containing mainly alpha cells (magenta). **(F)** Boxplots showing the mean percentage of beta or alpha cells per islet. Each dot represents the mean from a single slide. Scale bar: 100 μm.

To characterize pancreas tissue sections, thresholding detection and machine learning were used as described above (*Tissue, Islet and Cell Detection*) and applied to define the whole tissue as well as the exocrine and endocrine areas. For this purpose, six tissue sections from a non-diabetic donor were analyzed as shown in Table 1. Data regarding islet density, the number of cells per islet as well as their cellular composition (beta and alpha cells) were obtained (Figure 3 and Table 1). There were minimal differences in endocrine cell density, expressed as number of endocrine cells per islet area (mm^2^), between the sections (Figure 3A). Analysis of the cellular composition showed that the majority of islets contain between 10 and 100 endocrine cells (Figure 3B). As observed in Table 1, the mean area value of whole tissue, exocrine and endocrine compartments (including all slides) was 89.3 ± 19.7, 87.9 ± 19.2, and 1.3 ± 0.5 mm^2^ respectively. A similar number of islets was detected in the majority of tissue sections, even when individual pixel classifiers for each section were applied (283.7 ± 67.6 islets, Table 1). Only section #5, which had a bigger area, contained more islets than slide #1 to 4 and slide #6 (Figure 3B, Supplementary Figure S3 and Supplementary Table S3). There was a large variability in the proportion of alpha and beta cells per islet which ranged from 0 to 100% (Figures 3C, E and Supplementary Figure S4), as well as in endocrine cell density per islet (Figure 3D and Supplementary Figure S4). A mean of 70.1% of beta cells (range 57.7–79.9%) were present in the whole section versus a mean of 28.1% of alpha cells (range 20.1–33.3%) (Figure 3F). Cell density profiles showed a similar distribution for alpha and beta cells in different sections (Supplementary Figure S4).

### Assessment of the Reproducibility and Accuracy of Insulin (INS) and Proinsulin (PI) Positive Cell Detection

**FIGURE 4:**
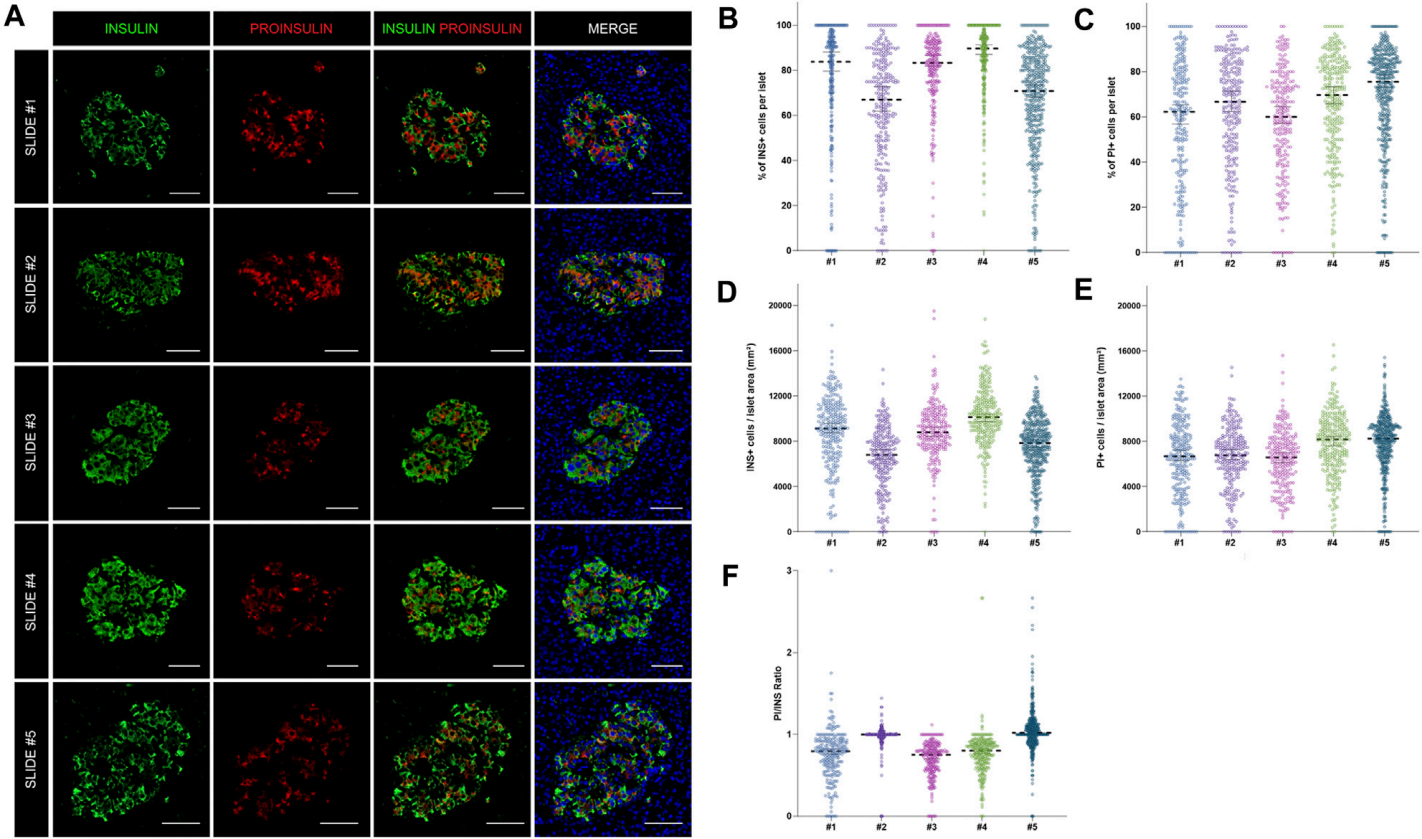
Assessment of the reproducibility and accuracy of insulin (INS) and proinsulin (PI) positive cell detection. **(A)** Representative images showing insulin (INS) and proinsulin (PI) staining in islets from different pancreatic sections stained with different antibody combinations (slides #1–5). **(B)** Dot plots showing the proportion of insulin and **(C)** proinsulin positive cells per islet in slides #1–5. Each dot represents an islet. **(D)** Dot plots showing the density of insulin and **(E)** proinsulin positive cells expressed as number of positive cells per islet area in mm^2^. Each dot represents an islet. **(F)** Comparison of the proinsulin to insulin ratio (PI/INS) among the different samples. Each dot represents an islet. Scale bar: 50 μm.

As shown above, insulin-producing beta cells are the predominant islet cell population. To further characterize them, the total number of cells positive for insulin (INS+) and proinsulin (PI+) was first measured in the whole tissue area of slides #1, #2, #3, #4, and #5 (Supplementary Table S2). The proportion of INS + cells was lower for sections #1, #2, and #5 (64.8, 56.2, and 53.5% respectively) compared to sections #3 (71.5%) and #4 (77.8%) (Supplementary Table S2). Slight differences between sections were expected: sections #1 and #2 were stained with a different insulin antibody and section #5 belonged to the same tissue block, but was not consecutive to the other slides (Supplementary Tables S2, S3). Conversely, the proportion of PI+ cells was comparable between sections, as the same antibody was used for all the slides (Supplementary Table S2). Then, the proportion and density of INS+ and PI+ cells per islet were calculated (Figure 4). Overall, there were mild differences in INS+ and PI+ cell distribution per islet between tissue sections.

As intraindividual differences could also be observed, the ratio PI/INS was calculated for each section (Figure 4F). Comparable results were obtained with median ratios that ranged from 0.79 to 1.02. In two sections (#2 and #3) the ratios were close to 1, indicating an equal detection of INS and PI in beta cells, whereas in other sections, lower ratios were observed. Therefore, mild variations in INS and PI expression from islet to islet and cell to cell are expected, even within the same individual.

### Analysis of the Proinsulin Processing Enzymes Prohormone Convertase 1/3, Prohormone Convertase 2 and Carboxypeptidase E

**FIGURE 5:**
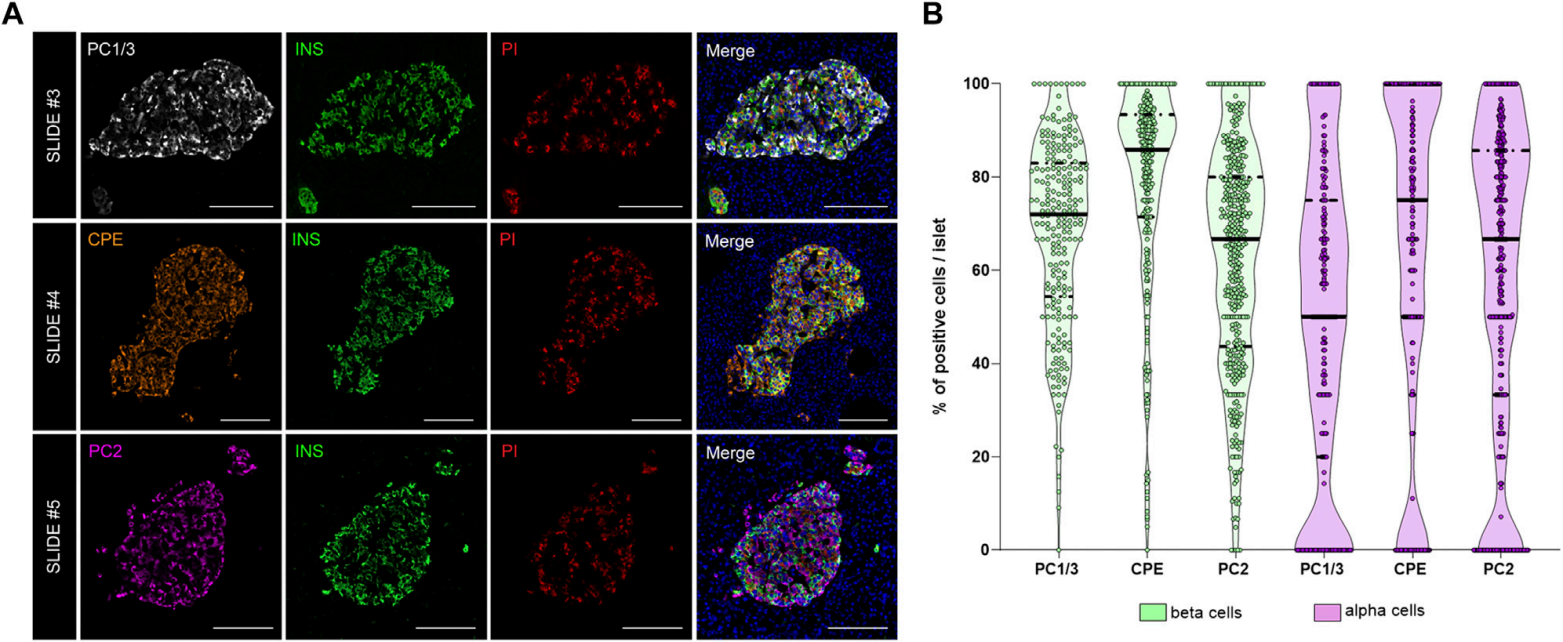
Analysis of the prohormone processing enzymes PC1/3, CPE and PC2 in the endocrine pancreas. **(A)** Representative images showing the distribution and localization of the proinsulin processing enzymes PC1/3 (slide #3, upper row), CPE (slide #4, middle row) and PC2 (slide #5, lower row) in the islets. **(B)** Violin plots showing the proportion of PC1/3+, PC2+, and CPE+ cells in beta (green) and alpha cells (violet). Each dot represents an islet. Scale bar: 100 μm.

As shown above, interindividual, inter-islet (among different islets of the same donor) and intra-islet (between cells within the same islet) differences in protein expression are expected. Therefore, establishment of reference expression levels for proteins like insulin, proinsulin, and their processing enzymes in non-diabetic individuals is of great interest. Thus, the expression of the enzymes PC1/3, PC2, and CPE was evaluated. The proportion of PC1/3+, PC2+, and CPE+ cells per islet was calculated in beta and alpha cells (Figure 5). Overall, the three prohormone enzymes were expressed in a higher percentage of beta cells compared to alpha cells, although there was high inter-islet variability (Figure 5B). CPE was expressed in a higher proportion of beta cells compared to PC1/3 and PC2 (mean CPE+ 78.5 ± 21.9%, mean PC1/3+ 68.2 ± 20.1%, and mean PC2+ 62.4 ± 24.1%). CPE and PC2 were expressed in a higher proportion of alpha cells compared to PC1/3 (mean PC1/3+ 49.3 ± 33.4%, mean PC2+ 58.4 ± 33.4%, and mean CPE+ 63.9 ± 35.9%).

### Analysis of the Proportion and Density of Immune Infiltration in the Exocrine and Endocrine Pancreas

**FIGURE 6:**
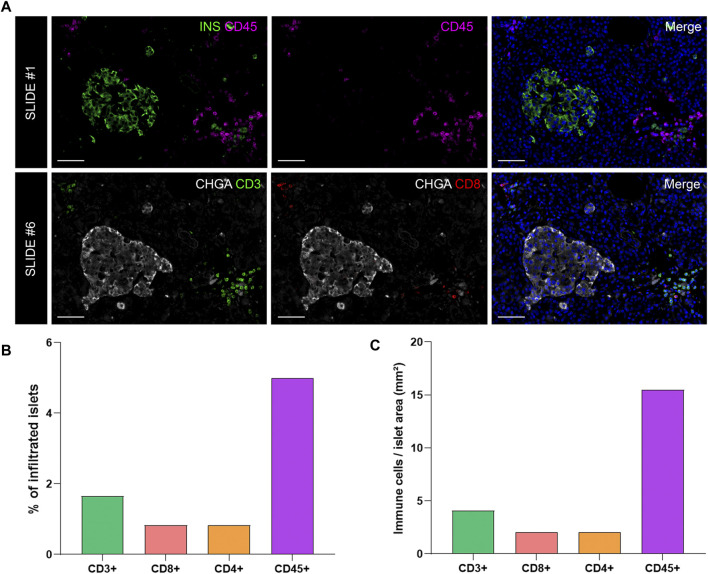
Analysis of the proportion and density of immune infiltration in the exocrine and endocrine pancreas. **(A)** Representative images of slide #1 showing CD45^+^ cells in the vicinity of an insulin-containing islet (upper row) and representative images of slide #6 showing CD3+ (lower row left) and CD8+ (lower row middle) cells close to a chromogranin A positive (CHGA+) islet. **(B)** Bar graphs show the proportion of infiltrated islets by CD3+, CD8+, CD4+, and CD45+ positive cells expressed as percentage of total islets. **(C)** Bar graphs show the density of CD3+, CD8+, CD4+, and CD45+ cells expressed as number of cells per islet area (mm^2^). Scale bar: 50 μm.

To investigate immune cell infiltration in the pancreas, cells were detected using the machine learning protocol described above (*Immune Cell Detection and Spatial Analysis of Immune Infiltration*). A minimum of 100 training points for each class of interest were assigned, and the proportion of CD45+, CD3+, CD8+, and CD4+ (CD3+CD8−) cells was calculated in the exocrine and the endocrine compartments using different sections from the same donor. As expected in a non-diabetic pancreas, there were no signs of insulitis, as currently defined. However, as observed in Figure 6, a few immune cells could be found close to, or infiltrating some islets. To evaluate immune infiltration in both compartments (exocrine and endocrine), the proportion of infiltrated islets, the proportion of immune cells, and immune cell density in the whole section were calculated (Figure 6 and Table 3). First, CD45+ cells were analyzed. In a total of 260 islets, only 18 CD45+ cells could be found within or immediately adjacent to islets (1% of the total number of CD45+ cells). Then, the proportion of CD3+, CD8+, and CD4+ cells per islet was calculated. In a total of 241 islets, 4 CD3+ cells (0.05%) could be found, of which two cells were CD8+ (0.05%) and two cells were CD8− (considered CD4+, 0.04%) (Table 3). The majority of immune cells were found in the exocrine tissue or in close proximity to blood vessels. Next, the proportion of islets that were infiltrated by at least one cell was calculated (Figure 6B). Only a few infiltrated islets were found in the whole section (5% by CD45+, 1.7% by CD3+, 0.8% by CD8+, and 0.8% by CD4+ cells).

Last, to evaluate the magnitude of the infiltration, T cell density was calculated as the number of infiltrating cells divided by the total exocrine or endocrine area (Table 3 and Figure 6C). As expected, density values were higher for CD45+ and CD3+ cells in both the exocrine and endocrine compartments (214.3 and 15.5 cells/mm^2^ for CD45+ cells; 116.7 and 4.1 cells/mm^2^ for CD3^+^ cells, respectively) while CD8+ (52.8 cells/mm^2^ in the exocrine and 2 cells/mm^2^ in the endocrine tissue) and CD4+ cell density (63.9 cells/mm^2^ in the exocrine and 2 cells/mm^2^ in the endocrine tissue) were lower.

### Two-Dimensional Spatial Analysis of the Localization and Distance of Immune Cells to the Islets

**FIGURE 7:**
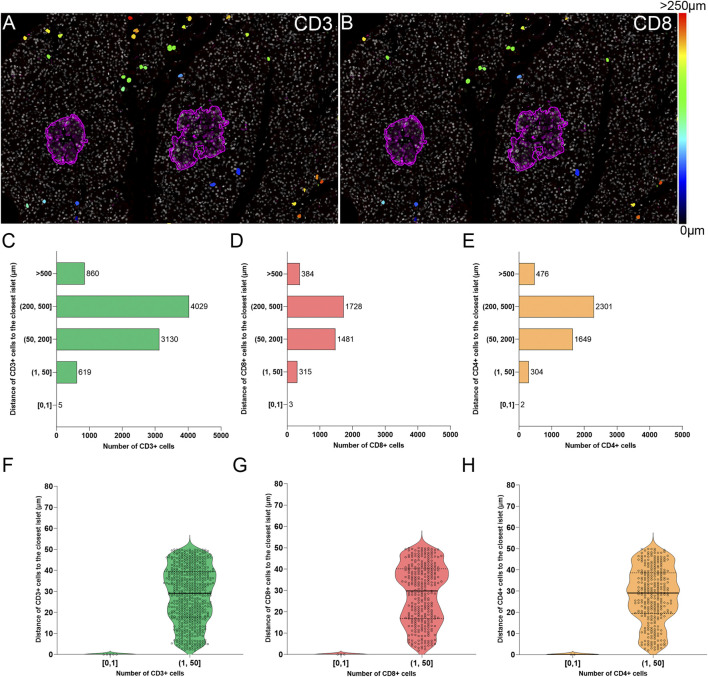
Two-dimensional spatial analysis of the localization and distance of immune cells to the islets. Heatmaps showing the distance of **(A)** CD3+ and **(B)** CD8+ cells to the closest islet. Scale: 0 (black-blue)—>250 (red) μm. **(C)** Bar graph showing the distribution of CD3+, **(D)** CD8+, and **(E)** CD4+ cells in five different distance categories to the closest islet: [0, 1), (1, 50], (50, 200], (200, 500], and >500 μm. **(F)** Violin plots showing the number of CD3+, **(G)** CD8+, and **(H)** CD4+ cells in the intra-islet [0, 1) and peri-islet (1, 50 μm]. Each dot represents a cell.

The current definition of insulitis takes into account both peri-islet (peri-insulitis), as well as intra-islet infiltration (intra-insulitis). Therefore, the location and distance of immune cells to the islets is an interesting feature for the analysis of immune infiltration in the context of T1D. As explained above (*Immune Cell Detection and Spatial Analysis of Immune Infiltration*), heatmaps were generated for CD3+ and CD8+ cells, which were color coded based on their distance to the closest islet. The majority of T cells were located far from islets while just a few cells were located close to islets (Figures 7A,B). Subsequently, the total number of CD3+CD8+ and CD3+CD8− (CD4+) T cells were grouped based on their distance to the islets (Figure 7). Five categories were defined: 1) between 0 and 1 μm; 2) between 1 and 50 μm; 3) between 50 and 200 μm; 4) between 200 and 500 μm, and 5) higher than 500 µm to the closest islet. The majority of T cells were found at a distance of 200–500 µm (4029 CD3+, 1728 CD8+, and 2301 CD4+ T cells). The distance range 1–50 µm represented the diameter of 3–5 acinar cells and it was considered the peri-islet area. For all T cell populations, a low number of cells was found in the periphery of the islets (619 CD3+, 315 CD8+, and 304 CD4+ T cells). As observed in Figures 7F–H, the number was low for cells infiltrating the islet parenchyma (distance of 0–1 µm: 5 CD3+, 3 CD8+, and 2 CD4+ T cells). This analysis revealed that under physiological conditions, immune cells can be found predominantly in the exocrine tissue at distances over 50 µm from the islets, whereas a low number of cells is located within and around the islet parenchyma.

## Discussion

The histological analysis of tissue sections has been historically challenging for pathologists due to time requirements, inter-observer variability, and the risk of biased interpretation (Aeffner et al., 2019). In T1D research, there is still a lot of ground to cover in deciphering the immunopathogenic mechanisms of the disease. In the last decade, the nPOD repository addressed the need for high quality human pancreatic specimens. Around the globe, researchers can now perform sophisticated multiplexed immunostainings, and acquire high-resolution whole-slide digitized images. This automatically creates a need for a quick, standardized and reproducible image analysis method. QuPath is an open-source software that enables whole-slide image analysis with time-saving and variability-reducing, semi-automated workflows (Bankhead et al., 2017). Interestingly, whole-slide image analysis is widely used in pancreatic malignancies for the detection of infiltrating immune cells or for annotation of tumor areas (Carstens et al., 2021; Zacarías-Fluck et al., 2021; Bulle et al., 2020), yet in the T1D research field this is still not a common practice. To date, out of the 552 publications citing QuPath (Bankhead, 2021), 27 used it for analysis of images from the pancreas or from isolated pancreatic islets (human or rodent): 24 were found related to pancreatic cancer, 3 to diabetes (Rajendran et al., 2020; Rubey et al., 2020; Apaolaza et al., 2021) and, to our knowledge, only 2 of them describe results from whole-image analysis of donors with T1D (Rajendran et al., 2020; Apaolaza et al., 2021).

Besides QuPath, whole-slide image analysis can be achieved with other open-source software, such as ImageJ [using the SlideJ plugin (Della Mea et al., 2017)], Orbit (Stritt et al., 2020), and Icy (de Chaumont et al., 2012). However, we are not aware of any studies comparing the reproducibility of the results, the time requirements for analysis or the ease of use among the three aforementioned software. HALO image analysis platform is a proprietary software from Indica Labs that can also handle whole-slide images; recently, scientists from the nPOD network analyzed whole-slide images from pancreata of non-diabetic, autoantibody-positive and T1D donors using the HALO platform, and reported alterations in the number and density of the acinar cells in donors with T1D (Tang et al., 2020). In a recent study, the reproducibility of the Ki67 measurement and the subsequent predictability of cancer prognosis were compared among three image analysis platforms: HALO, QuantCenter (from 3D Histech), and QuPath. While Ki67 scoring can prove useful for the prediction of cancer prognosis, the variability in pre-analytical, analytical (experimental), and especially in manual scoring protocols has discouraged pathologists from implementing it in the clinical practice. Conversely, using the mentioned Image analysis platforms yielded excellent results. Ki67 scoring and prognosis predictability were “indistinguishable” among the three platforms even when different operators were employed, thus urging scientists to opt for automated analysis solutions, in order to avoid the variability of manual analysis and thus accelerate the implementation of digital Ki67 scoring in the clinic (Acs et al., 2019).

In this study, we used intensity thresholding, pixel classification and machine learning algorithms in QuPath to precisely and automatically detect different structures in the pancreas from multiplexed immunofluorescence images regardless the staining protocols. We were able to run an accurate anatomical (tissue size, islet areas, etc.) and physiological characterization (insulin, proinsulin, and prohormone enzyme profiles) of whole pancreas sections from a non-diabetic donor, establishing an image analysis pipeline that can be applied not only to the study of T1D but also to other diseases of the pancreas. Importantly, we found similar islet numbers and densities, as well as similar distribution for alpha and beta cells between sections, demonstrating the validity of the parameters implemented in our protocols. In line with previous studies (Steiner et al., 2010; Dolenšek et al., 2015; Da Silva Xavier, 2018; Noguchi and Huising, 2019), we confirmed that insulin-producing beta cells constitute 60–70% of the islet cell population, whereas around 30–40% are alpha cells. In addition, we showed that in a non-diabetic condition the majority of the islets contain between 10 and 100 endocrine cells. This parameter is worth to be considered, as differences in the number of endocrine cells forming the islets can also indicate beta cell decay. Independently of the antibody combination used, the median values for INS+ and PI+ cells, as well as the PI/INS ratio were comparable, with few exceptions.

Furthermore, changes in the expression and distribution of the proinsulin processing enzymes can indicate failure of these specific prohormone conversion mechanisms. Differences may exist even in non-diabetic individuals, which can be extended to high intra-individual or even intra- and inter-islet heterogeneity under pathogenic conditions (Teitelman, 2019). Proteomic analysis of islets obtained by laser capture microdissection (LCM) indicated that PC1/3 and CPE are reduced in islets from donors with T1D with long disease duration (Wasserfall et al., 2017; Sims et al., 2019). Impaired proinsulin conversion accompanied by elevated proinsulin secretion is characteristic of T2D and T1D, and defects in proinsulin processing result in alteration of the PI/C-peptide and PI/INS ratios (Sims et al., 2019; Sims et al., 2019). Of note, there has been some controversy regarding the role of PC2 in proinsulin processing in humans; a recent paper, Ramzy and colleagues (Ramzy et al., 2020) provide evidence that PC2 is neither abundant nor plays a significant role in the processing of proinsulin in human beta cells, whereas other groups have reported the abundancy of PC2 in human pancreata (Scopsi et al., 1995; Teitelman, 2019). However, a limitation of these analyses is that they were not performed in whole pancreas sections, thus capturing the majority of islet types in an individual, but in isolated islets and beta cell lines. Here, we provide an analysis pipeline to estimate the proportion and density of beta and alpha cells, as well as of the processing enzymes PC1/3, PC2, and CPE in the islets or endocrine compartment, which could uncover important alterations in insulin production under inflammatory or stressful conditions and provide comprehensive evidence to fundamental mechanistic questions of proinsulin processing.

Modern lifestyle and diet have placed an enormous amount of metabolic pressure on beta cells, which are constantly hyper-functioning to produce and secrete insulin. This metabolic stress could lead to mistakes in the translational and post-translational processing of insulin and other beta cell proteins, which could in turn lead to the generation of neoantigens and to the ultimate recruitment of the immune cells to the pancreas (Rodriguez-Calvo et al., 2021). Insulitis is a hallmark of T1D; CD8+ T cells are the most abundant cell population in an insulitic lesion, followed by CD68+ macrophages, CD20+ B cells, and CD4+ T cells (Willcox et al., 2009). Even though insulitis seems to matter most because of the consequent destruction of beta cells and the loss of insulin, it has been shown that the exocrine compartment is also infiltrated by CD8+, CD4+, and CD11c+ cells (Rodriguez-Calvo et al., 2014; Campbell-Thompson et al., 2015). Despite these observations, the immunopathological course from health to disease, as well as the importance of the crosstalk between the endocrine and the exocrine tissue are still unclear. The detection of T cells around or within the islets, as well as their dynamic distribution in the endocrine and the exocrine pancreas are of great interest. Here, we described two ways of detecting immune cells using QuPath: 1) using the *single measurement classifier* based on thresholding or 2) by *machine learning*. Our results show that the machine learning option is quicker and more accurate and we recommend its use for the detection of infiltrating immune cells in the pancreas. In this study, we have evaluated their number, density and distance to the islets. Distance-wise, the majority of the T cells are found between 50 and 500 µm away from the closest islet in a non-diabetic pancreas. However, as disease progresses, a higher number of immune cells might be found closer to or inside the islets. This type of analysis can help to understand the dynamics of immune infiltration in the pancreas in individuals with prediabetes, as well as after onset of disease, and could inform clinical trials aiming to halt the autoimmune attack in T1D (Herold et al., 2019). As reported recently (Berben et al., 2020), the semi-automated methods offered by QuPath are equally reliable and considerably quicker than manual counting of immune infiltrating cells—a method that is still considered the gold standard in the clinical setting.

From a practical point of view, the working time with QuPath ranges between 1.5 and 2 h per slide, depending on computer processing power, memory and user’s experience level. Most of this time is devoted to finding the correct settings in cell detection and intensity thresholds for each channel that will work for different sections. In our experience, these two parameters can strongly influence cell segmentation and subsequent positive or negative identification of cells for markers of interest. Thus, we advise researchers to test these parameters in small areas, and in different types of donor sections (control, disease, etc), in order to find which ones will work for most, if not all, types of samples. Despite slowing the process at the beginning of the analysis, the aforementioned preliminary testing will save time during the actual analysis, and prevent the occurrence of segmentation or detection issues among the different types of samples. Besides the actual working time with QuPath, the user should plan time for data-processing, grouping, and analysis, which depending on the number of desired readouts, may range from 2 to 5 h per slide. Overall, the time invested in whole-slide image analysis yields high quality data and we hope that, together with the step-wise guide provided here, it will encourage the performance of large-scale image analysis studies.

We believe that researchers should take advantage of the increasingly available digitized whole-slide pancreatic images and of the numerous open-source tools offered by QuPath. Taken together, we established several image analysis workflows that provide a basic guide to improve the characterization of the exocrine and endocrine compartments, islet cell populations, and immune infiltration. We acknowledge that other methods and analytical tools within QuPath could be used to obtain similar datasets, and that these should be customized based on quality of the sample, staining parameters and analytical goals. In addition, intra-individual variability should be assessed, as there are several factors that could contribute to it: 1) the use of different antibody combinations and/or protocols on different sections; 2) even though some sections might be consecutive, it does not necessarily mean that all the sections contain the same islets or cells; 3) stainings might not be performed on the same day, and this could add inter-staining variability. The fact that we were able to detect and quantify the extent of this variability through the use of QuPath makes us more confident that we provide an objective workflow for large-scale studies. Moreover, samples from different donor groups and disease status need to be included in the first steps of the image analysis workflow to assess inter-donor variability and to ensure that all the parameters are applicable to the different experimental conditions. Therefore, we invite other scientists to share their image analysis pipelines with the scientific community to maximize the impact of open-access tools. Here, we provide an analysis pipeline customized for the analysis of pancreas specimens with the aim of improving the accuracy, reproducibility and objectivity of image analysis while shortening the analysis time. These tools should help to gain new insights into the pathogenesis of diabetes and other pancreatic diseases, and could accelerate research on biomarker discovery and pharmacological interventions aimed at the diagnosis and cure of T1D.

## Data Availability

The original contributions presented in the study are included in the article/Supplementary Material, further inquiries can be directed to the corresponding author.
