# Supplementary figures and images for: Whole-Slide Image Analysis of Human Pancreas Samples to Elucidate the Immunopathogenesis of Type 1 Diabetes Using the QuPath Software
Source: Front Mol Biosci. 2021 Jun 11;8:689799. doi: 10.3389/fmolb.2021.689799 (PMC8226255; doi:10.3389/fmolb.2021.689799)

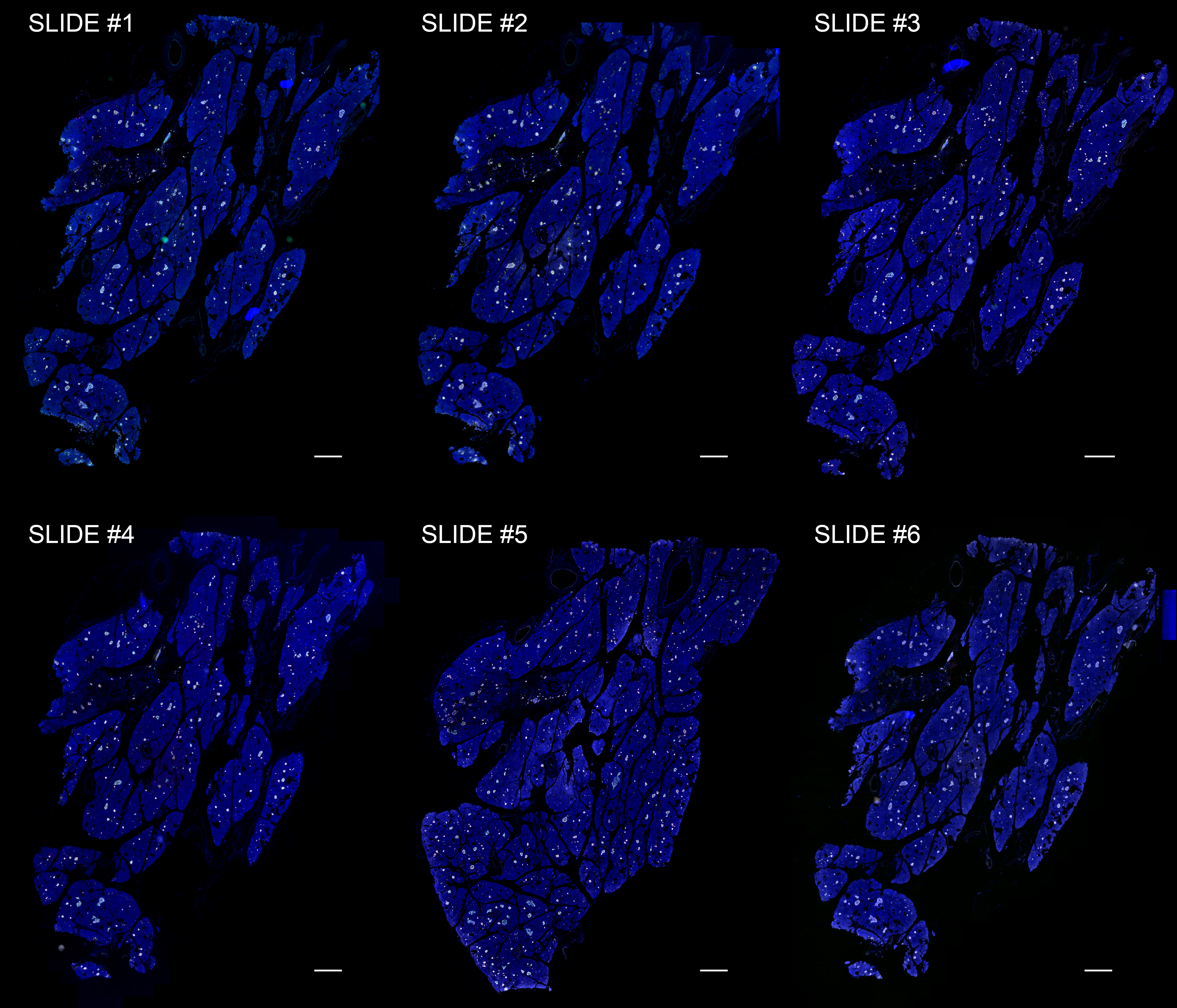

Supplement: Supplementary file 1 [file Image3.JPEG]

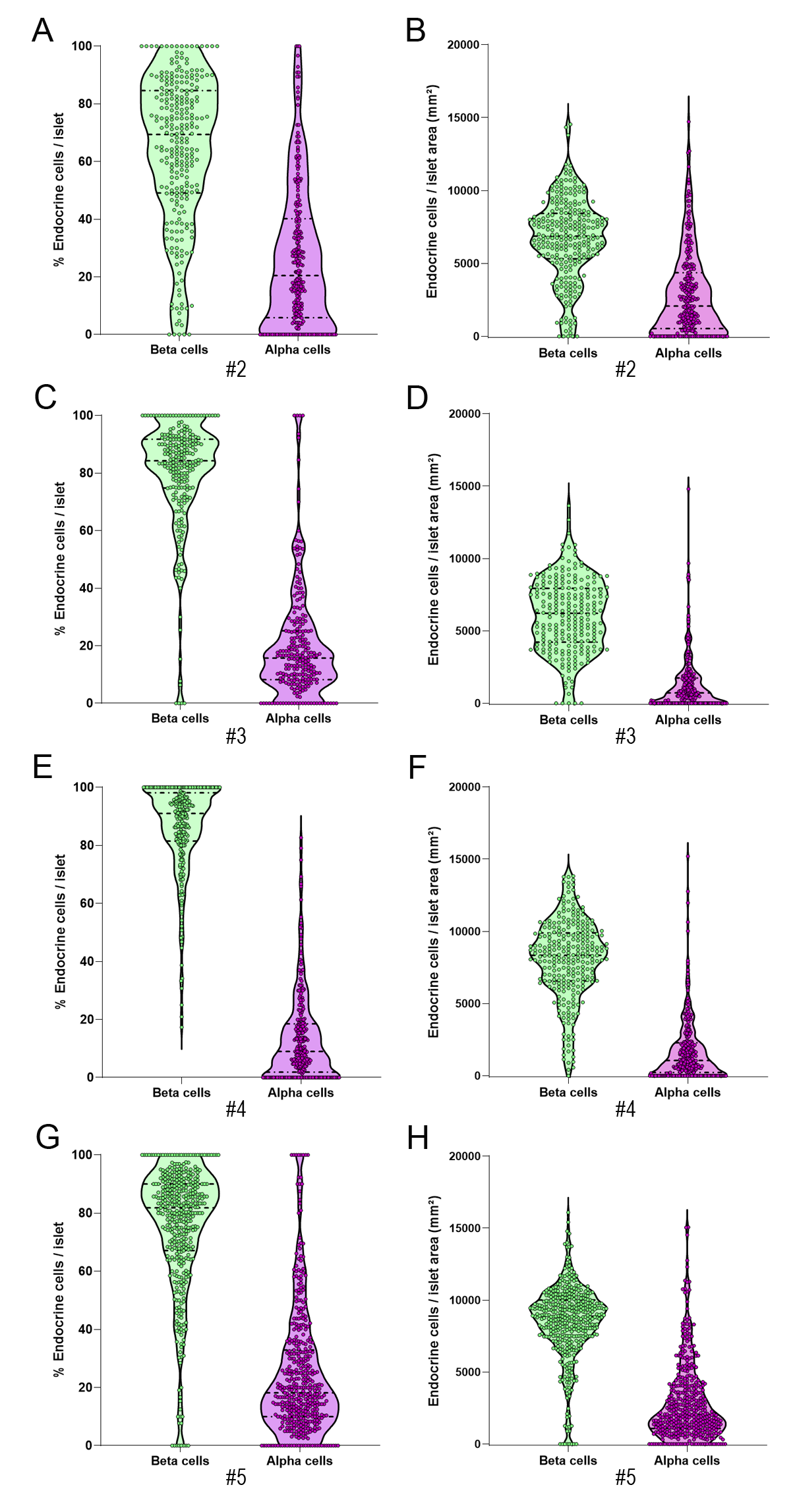

Supplement: Supplementary file 3 [file Image4.TIF]

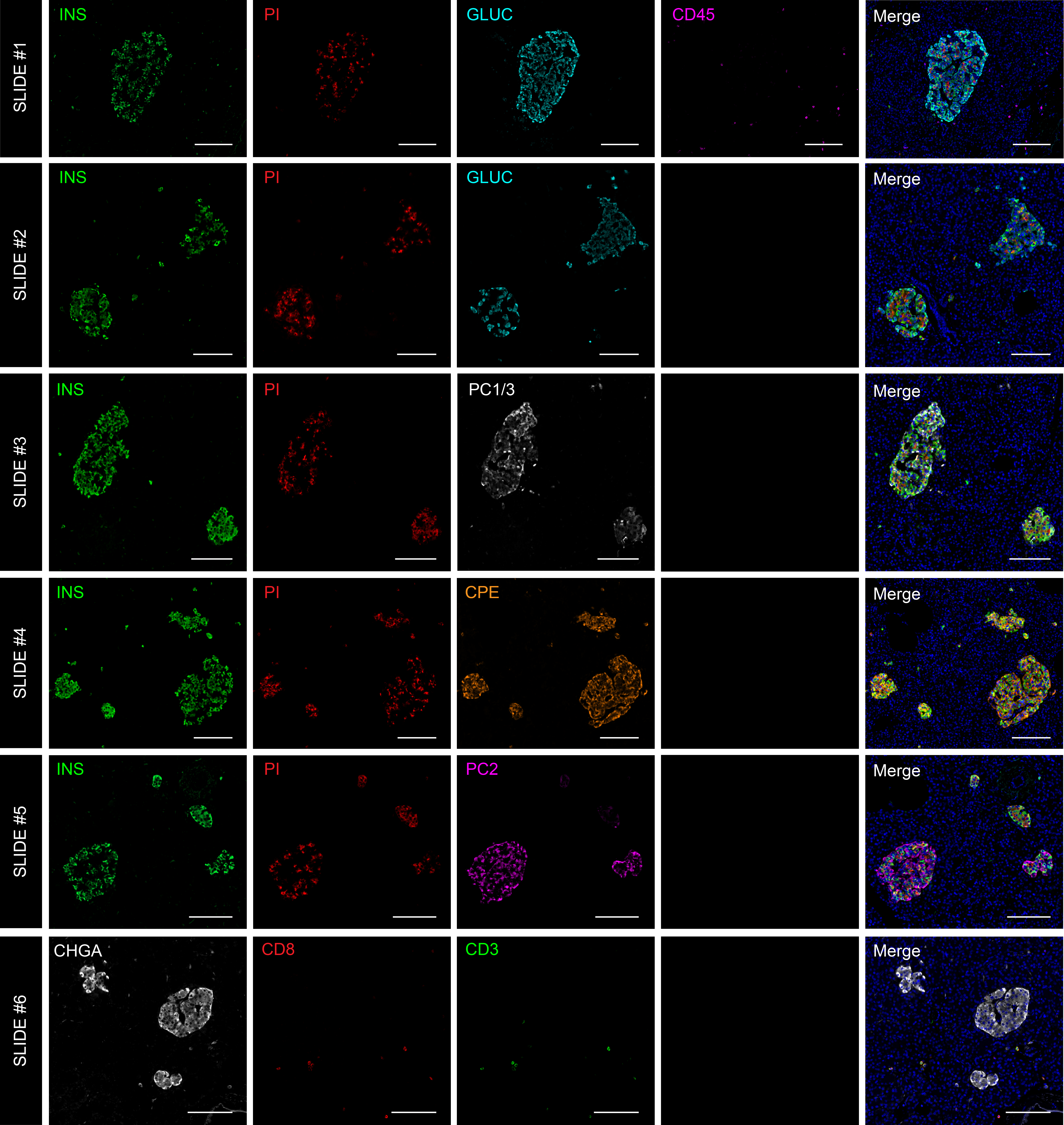

Supplement: Supplementary file 4 [file Image1.JPEG]

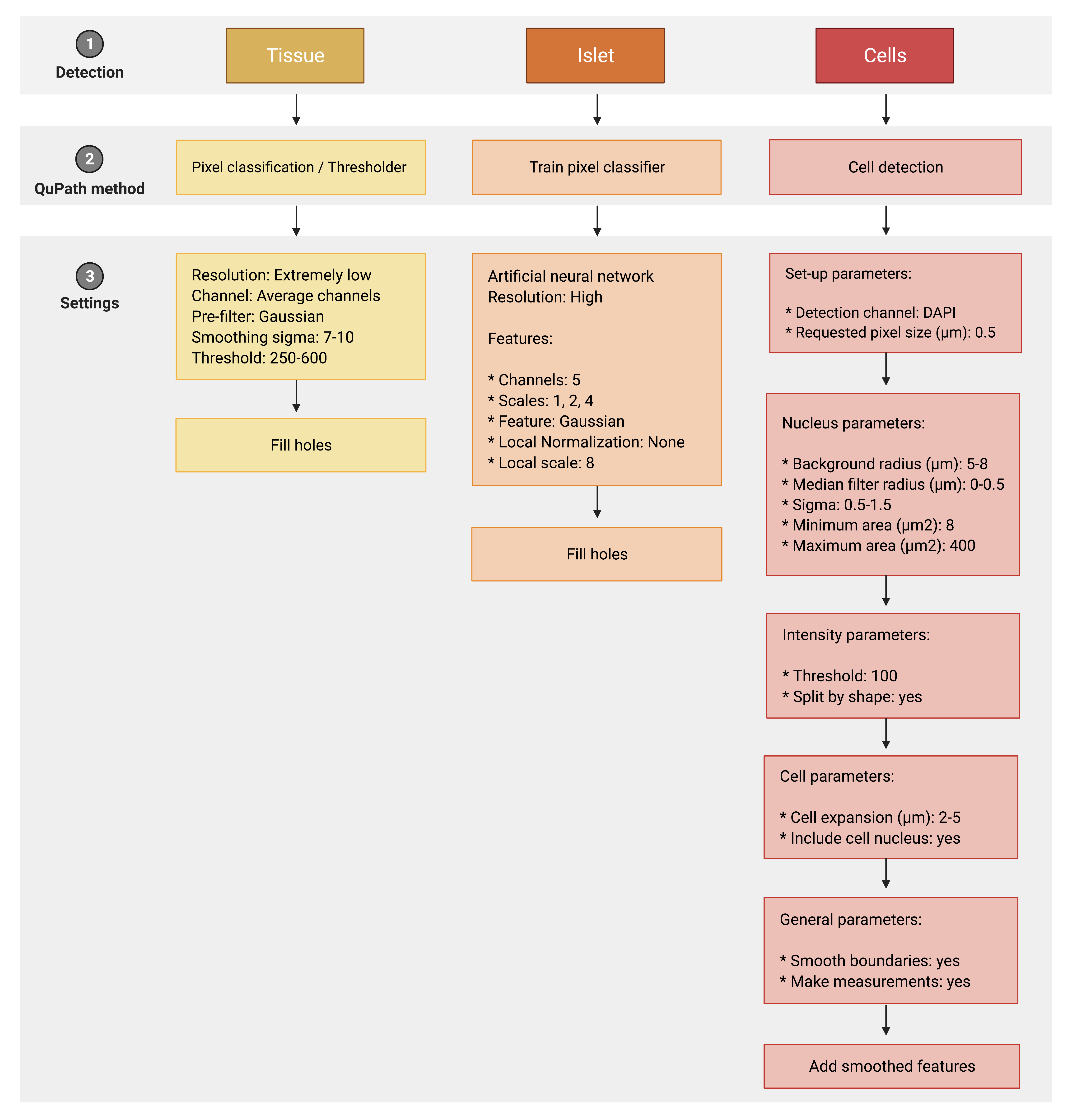

Supplement: Supplementary file 6 [file Image2.PNG]
