# Supplementary material for: Whole-Slide Image Analysis of Human Pancreas Samples to Elucidate the Immunopathogenesis of Type 1 Diabetes Using the QuPath Software
Source: Front Mol Biosci. 2021 Jun 11;8:689799. doi: 10.3389/fmolb.2021.689799 (PMC8226255; doi:10.3389/fmolb.2021.689799)
Supplement: Supplementary file 2 [file Table1.docx]

| Staining | Primary Antibody | | Clone | Source | Dilution | Secondary Antibody | Dilution |
| --- | --- | --- | --- | --- | --- | --- | --- |
|  | | Anti-Human Proinsulin | SP2/0-Ag14 | DSHB - #GS-9A8 | 1:200 | opal 570 | 1:100 |
| 1-PI/CD45/INS/GCG | | Anti-Human CD45  Anti-Human Insulin  Anti-Human Glucagon | 2B11+ PD7/26  polyclonal  EP3070 | Agilent Tech. - #M070101  Agilent Tech - #A056401-2 (discontinued)  Abcam - #ab92517 | 1:100  1:500  1:1200 | opal 650  goat anti-guinea pig AF488  goat anti-rabbit AF750 | 1:100  1:1000  1:1000 |
|  | | Anti-Human Insulin | polyclonal | Agilent Tech - #A056401-2 (discontinued) | 1:500 | goat anti-guinea pig AF488 | 1:1000 |
| 2-INS/PI/GCG | | Anti-Human Proinsulin | SP2/0-Ag14 | DSHB - #GS-9A8 | 1:200 | goat anti-mouse IgG1 AF555 | 1:1000 |
|  | | Anti-Human Glucagon | EP3070 | Abcam - ab92517 | 1:1200 | goat anti-rabbit AF750 | 1:1000 |
|  | | Anti-Human Insulin | MAB1 | Merck Millipore - #05-1108-KC | 1:300 | opal 520 | 1:200 |
| 3-INS/PC1/PI | | Anti-Human PCSK1N | polyclonal | Atlas Ant. - #HPA064734-100 | 1:500 | goat anti-rabbit AF555 | 1:1000 |
|  | | Anti-Human Proinsulin | SP2/0-Ag14 | DSHB - #GS-9A8 | 1:300 | goat anti-mouse IgG1 AF647 | 1:1000 |
| 4-INS/CPE/PI | | Anti-Human Insulin  Anti-Human CPE  Anti-Human Proinsulin | MAB1  polyclonal  SP2/0-Ag14 | Merck Millipore - #05-1108-KC  Atlas Ant. - #HPA003819  DSHB - #GS-9A8 | 1:300  1:100  1:200 | opal 520  goat anti-rabbit AF555  goat anti-mouse IgG1 AF647 | 1:200  1:1000  1:1000 |
| 5-INS/PC2/PI | | Anti-Human Insulin  Anti-Human PC2  Anti-Human Proinsulin | MAB1  polyclonal  SP2/0-Ag14 | Merck Millipore - #05-1108-KC  Merck Millipore - #AB15610  DSHB - #GS-9A8 | 1:300  1:800  1:300 | opal 520  goat anti-rabbit AF555  goat anti-mouse IgG1 AF647 | 1:200  1:1000  1:1000 |
| 6-CD3/CD8/CHGA | | Anti-Human CD3  Anti-Human CD8A  Anti-Human Chromogranin-A | polyclonal  polyclonal  polyclonal | Agilent Tech. - A045229-2  Atlas Ant. - #HPA037756  Abcam - #ab15160 | 1:200  1:900  1:500 | opal 570  opal 650  goat anti-rabbit AF488 | 1:200  1:200  1:1000 |
| * A 2-step microwave antigen retrieval process preceded the primary antibody incubations, and was the same for all the stainings. | | | | | | | |

**Table S1:** Information on the staining combinations, primary antibody, clone, source and its dilution, and secondary antibody and its dilution.

**Table S2:** Comparison of the number, proportion and density of total insulin (INS+) and proinsulin (PI+) positive cells among the sections stained with different antibody combinations.

|  | **Total number of positive cells** | | **Proportion (%)** | | **Cell density (cells/mm²)** | |
| --- | --- | --- | --- | --- | --- | --- |
| Sample ID | INS+ | PI+ | INS+ | PI+ | INS+ | PI+ |
| 1-PI/CD45/INS/GCG | 8832 | 6439 | 64.8 | 47.3 | 7599.5 | 5540.5 |
| 2-INS/PI/GCG | 7383 | 7328 | 56.2 | 55.8 | 5917.9 | 5873.9 |
| 3-INS/PC1/PI | 8900 | 5658 | 71.5 | 45.5 | 8025.4 | 5101.9 |
| 4-INS/CPE/PI | 11373 | 8196 | 77.8 | 56.1 | 9177.8 | 6614.0 |
| 5-INS/PC2/PI | 14270 | 15760 | 53.5 | 59.1 | 6192.4 | 6839.0 |
| Mean ± SD | 10151.6 ± 2425.9 | 8676.2 ± 3642.6 | 64.8 ± 9.1 | 52.8 ± 5.4 | 5938.1 ± 2817.6 | 5993.9 ± 650.3 |

**Table S3:** Comparison of tissue, endocrine and the exocrine area, number of islets, cells and beta and alpha cells from: all the slides (total mean ± SD); slides #1, 2, 3 and 4 (mean ± SD), and slide #5 (mean). Slides #1, 2, 3 and 4 were consecutive, while slide #5 was obtained from the same block, but it was not consecutive to the previous slides.

|  | **Tissue Area (mm²)** | **Exocrine Area (mm²)** | **Endocrine Area (mm²)** | **n° islets** | **n° islet cells** | **n° beta cells** | **n° alpha cells** |
| --- | --- | --- | --- | --- | --- | --- | --- |
| Total mean ± SD | 89.3 ± 19.7 | 87.9 ± 19.2 | 1.3 ± 0.5 | 283.7 ± 67.6 | 15611.5 ± 4981.8 | 11090.6 ± 3500.9 | 4822.2 ± 2199.8 |
| Slides #1, #2, #3, #4 mean ± SD | 81.0 ± 7.4 | 79.9 ± 7.4 | 1.1 ± 0.1 | 254.2 ± 14.2 | 13402.8 ± 715.7 | 9468.5 ± 1471.1 | 3758.8 ± 628 |
| Slide #5 mean | 130.6 | 128.3 | 2.3 | 432 | 26655 | 17579 | 9076 |
