# Supplementary material for: Whole-Slide Image Analysis of Human Pancreas Samples to Elucidate the Immunopathogenesis of Type 1 Diabetes Using the QuPath Software
Source: Front Mol Biosci. 2021 Jun 11;8:689799. doi: 10.3389/fmolb.2021.689799 (PMC8226255; doi:10.3389/fmolb.2021.689799)
Supplement: Supplementary file 5 [file DataSheet1.PDF]

Standard Operating Procedure for whole-slide image analysis of pancreatic sections using the open-source software QuPath (version 0.2.3)

For more detailed information, contact teresa.rodriguez@helmholtz-muenchen.de.

For further instructions and basic information go to: https://qupath.readthedocs.io/en/latest/.

1. Create a new project:

- Create project → Create an empty folder with the name of the project
- *Add images* → *Set image type*: *Fluorescence* → *Choose files* or drag and drop → Import.

2. Image preparation:

- Double click on the image of interest.

  - Adjust intensity levels or change the color of the channel by double clicking on the channel name.
  - If z-stacks are available, try to find the best focus and work on that slice. See the image below for examples (the middle slice was better focused than the lower and higher one).

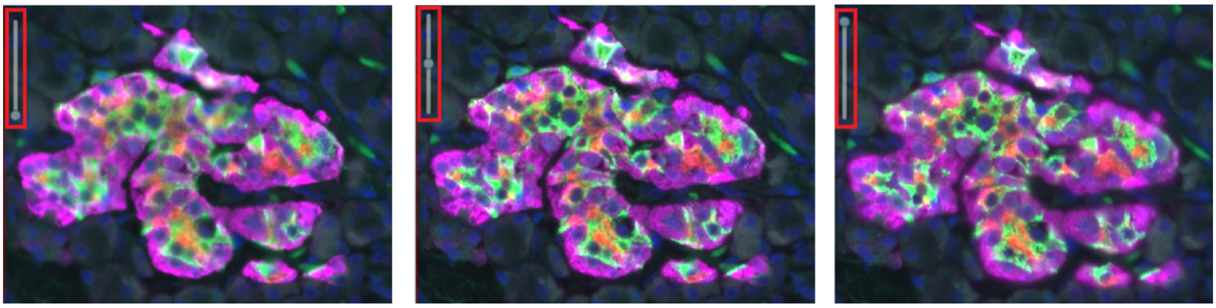

  - If the maximum display (red rectangle below) is too low, modify it by double clicking on the number. See below for example:

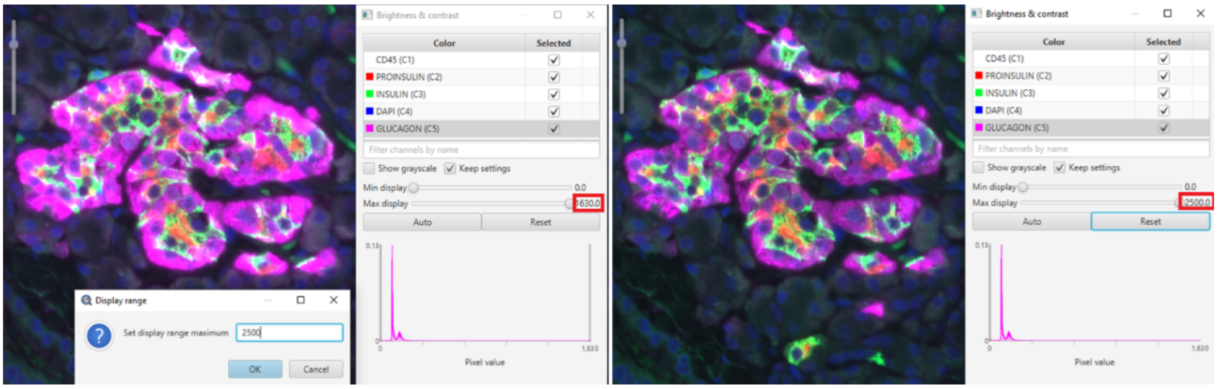

  - The image is now ready to be analysed.

3. Tissue detection:

- Can be performed before or after Islet detection.
- Create a class called *Tissue*.
- Go to *Classify* → *Pixel classification* → *Create thresholder*
- Settings:

  - Chose the desired resolution and DAPI as channel. The Gaussian prefilter is usually preferred.
  - Smoothing sigma: higher values than 0 create a more homogeneous area. *High values will overestimate the tissue area.
  - The threshold will depend on the intensity of the staining for DAPI.
  - Chose Everywhere for the region.
  - Give a name and save the new classifier.

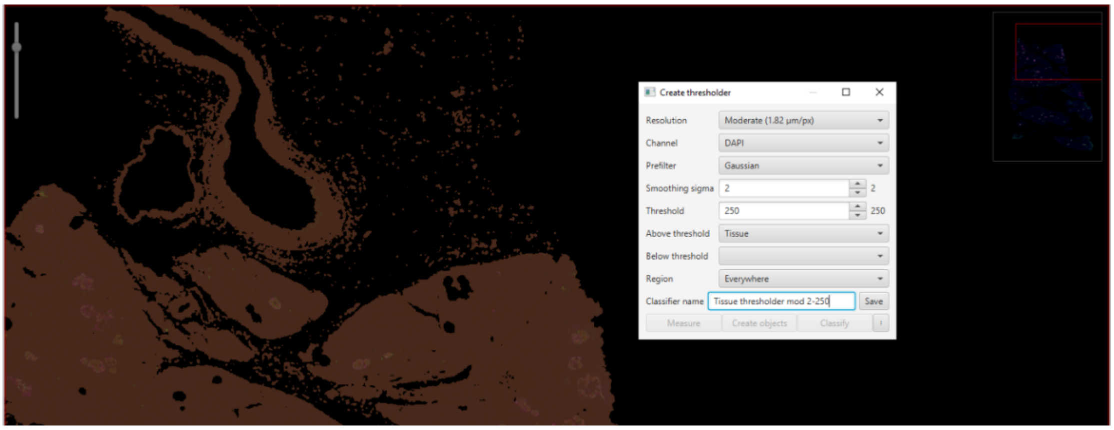

  - Once it is saved, go to *Create objects*.
  - A new window appears: "Pixel classifier". Chose *Parent objects* → *Select Full image.*
  - A window to create objects appears: In this case the minimum object size should be bigger than the minimum hole size.
  - Deselect *Split objects*: The tissue should be annotated as a single object.
  - A tissue area is now created for each z-stack. Select the one with the best focus.
  - Delete the annotations of the other stacks.

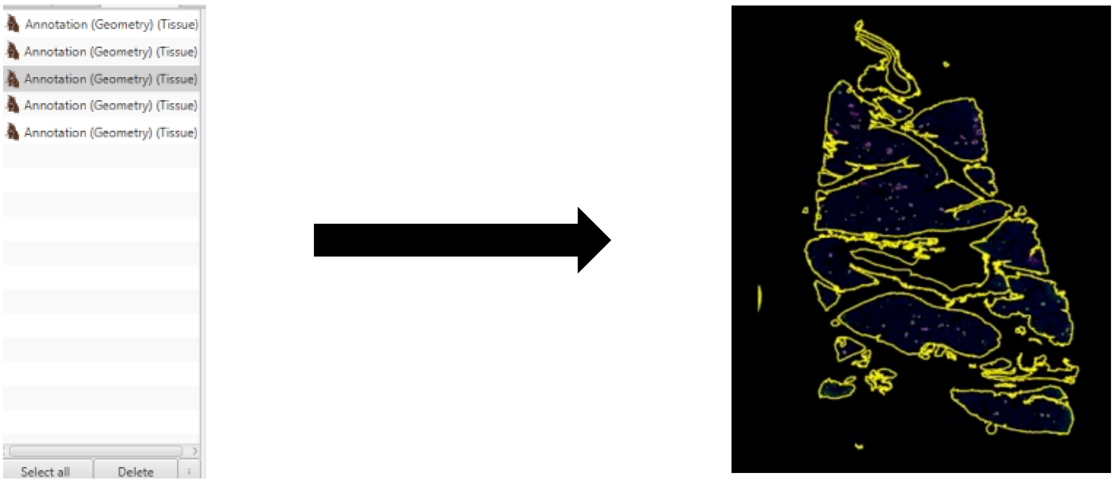

- Manual correction might need to be done and certain areas of the detected tissue can be erased using the brush tool and by pressing Alt+left click. Check the QuPath website for more information: https://qupath.readthedocs.io/en/latest/.

4. Islet detection:

- Work on the z-stack with the best focus.
- Create a new class called *Islet*.
- If the exocrine compartment is not important for subsequent analysis, the classes *ignore** and *islet* can be used. However, an *Exocrine* class can be created and used instead of **ignore*, if the exocrine compartment will be included in the analysis.
- Start drawing areas for each class and press *Set class.*
- Go to *classify* → *Pixel classification* → *Train pixel classifier.*

- Settings for the Pixel classifier:

  - Inside the menu *Default multiscale features*: Choose the channels that are relevant for the detection of the islets. Select a few scales to adjust the amount of smoothing. Chose the Gaussian feature that is given by default and avoid local normalization.

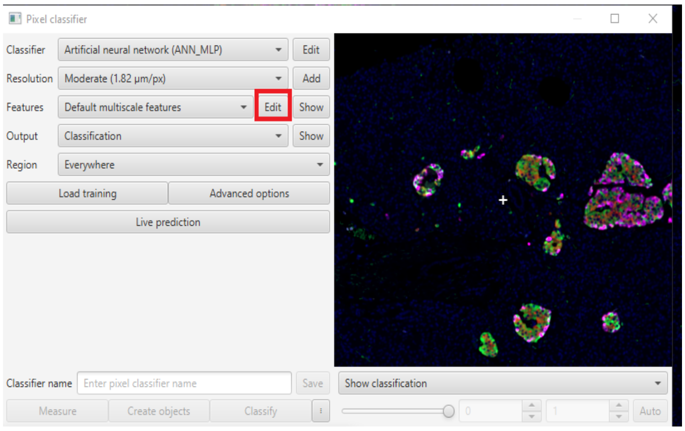

- Press *Live prediction*, and do not forget to press 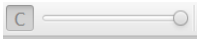 in order to see the pixel classifier.
- When detection is satisfactory, save the classifier.
- Check if the detection is correct by creating objects. Test it in a new small area first (e.g., in a rectangle). Select the area and then press *Create objects* → *Choose parent objects*: *Current selection*→OK.
- For islets, it is recommended to go to the image, find the smallest islets of interest and check their area. Then select a minimum size in µm².
- Mark *Split objects*.
- New objects are now classified as islets.
- Some islets can present holes inside that need to be filled:

  - First select all the islets or objects. Left panel → *Select all* (afterwards you can deselect the main region or tissue area).

- *Objects → Annotations → Fill holes.*

5. Cell detection:

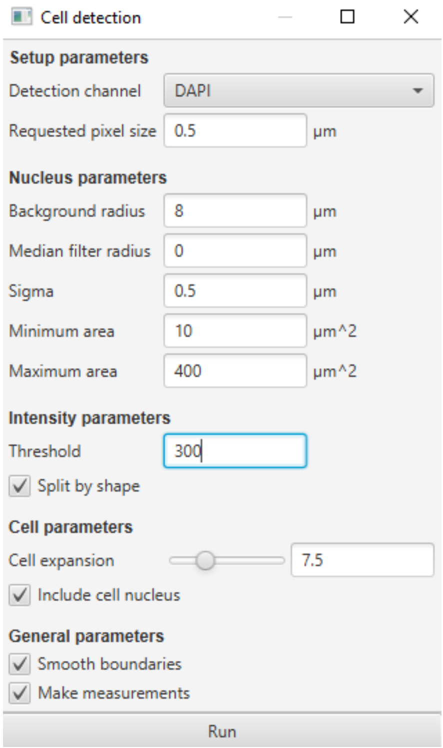

- To find the right settings, first draw a new small area. Keep the annotation selected every time you run the cell detection.
- Run cell detection once with the default values and correct the settings afterwards, if necessary.
- Go to *Analyze → Cell detection → Cell detection.*
- These options must be selected for visualization 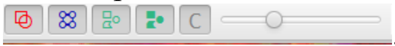.
- <u>Median filter</u> reduces texture (increasing the value might create spiky cell nuclei).
- <u>Sigma value</u> is 1.5 by default. Lower values increase the number of detected cells (also increases the chances of divided nuclei).
- <u>Minimum area</u> and <u>maximum area</u> usually work well with the default settings.
- <u>Threshold</u>: Check the cell or nucleus mean intensity values for DAPI.

Threshold value: 100 Threshold value: 300

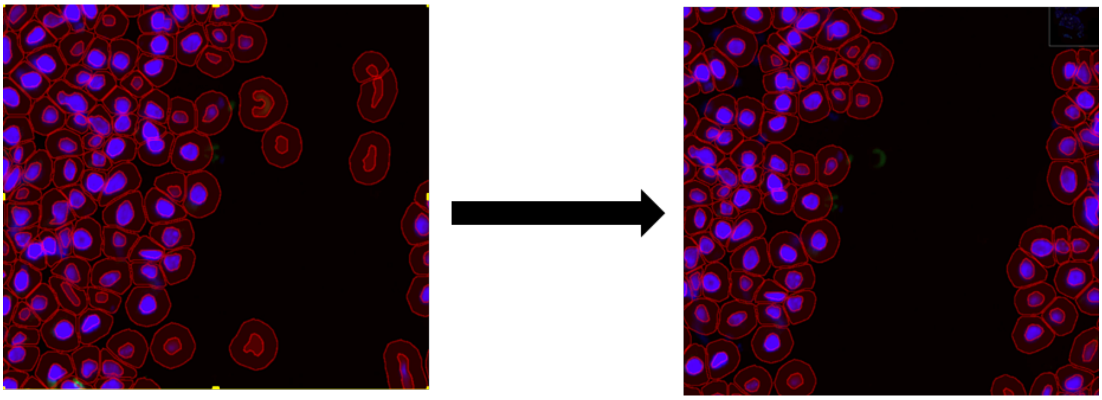

- <u>Cell expansion</u>: Cell expansion is a critical parameter that determines the size of the detected cells. In our analyses we have found that a cell expansion of 7.5-10 µm works best for islet cells. For immune cells an expansion of 2-5 µm would be more appropriate (if these were the only cell population to be analyzed). A test in a small rectangle area where all the markers of interest are located is advised.
- <u>Split by shape</u>, <u>include nucleus</u> and <u>general parameters</u> must be always selected.
- Select the tissue annotation and run *Cell detection* with the chosen settings.
- Go to *Analyze → Calculate features → Add smoothed features*. QuPath will create additional measurements considering the characteristics of the neighbour cells in a radius of 25µm.

6. **Analysis of proinsulin, insulin and glucagon by thresholding:**

- If it has not been done before, apply the islet pixel classifier on the tissue annotation and create the objects as explained above.
- Create a class for every protein of interest.
- Go to *Classify* → *Object classification* → *Create single measurement classifier*.
- A classifier for each protein has to be created (channel filter).
- Select the <u>channel filter</u>: Proinsulin, insulin or glucagon.

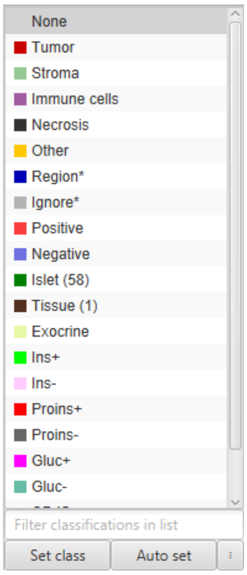

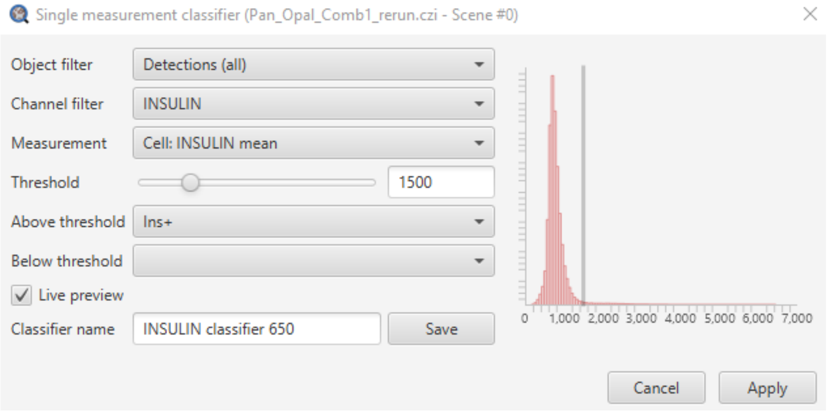

- <u>Measurement</u>: Try *Cell mean*, *Cytoplasm mean*, *Smoothed cell mean* or *Smoothed cytoplasm mean*. Move the threshold to find the best fit. * Try to set a high threshold that still allows you to detect the positive cells. A lower threshold will detect more cells, but it will result in many double or triple positive cells (which are not truly double or triple positive) when combined with the classifiers for the other markers. Furthermore, the detection of positive cells is highly dependable on the cell segmentation, thus it is extremely important to select the right parameters at cell detection.
- For better visualization, select only the channel you want to see in *brightness & contrast*, and increase the opacity of the *cell & boundaries* to see the color of the class.
- Click *live preview*.
- This is how it should look:

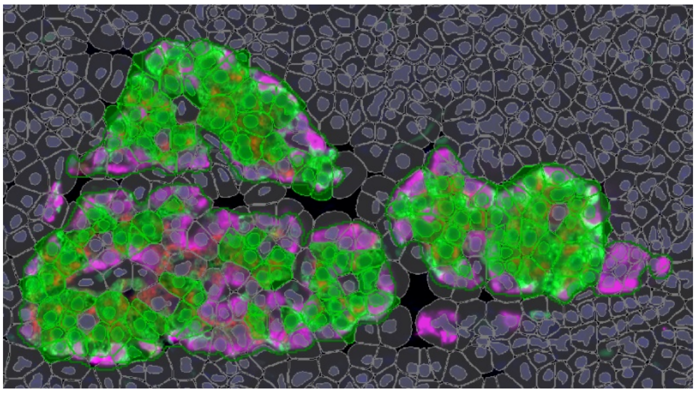

- Save the classifier.
- Repeat the same for insulin and glucagon.

- Once the three single classifiers are created, it is time to combine them: Go to *Classify* → *Object classification* → *Create composite classifier*.

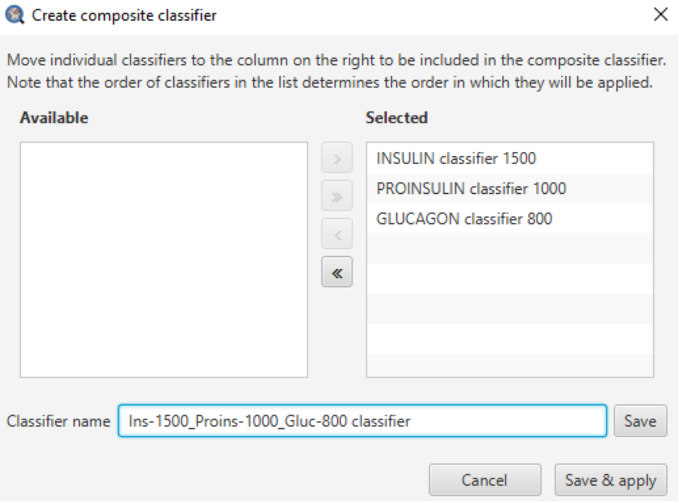

- Give a name to the combine classifier and apply.
- Select *centroids only* or *boundaries only* to see the detections defined for example as Proins+Ins+Glu+ with a specific symbol or color.

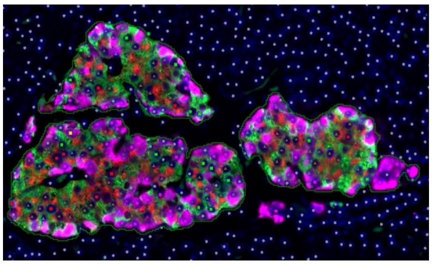

Green square: Ins+

Red square: Proins+

Magenta square: Gluc+

Olive triangle: Ins+ Proins+

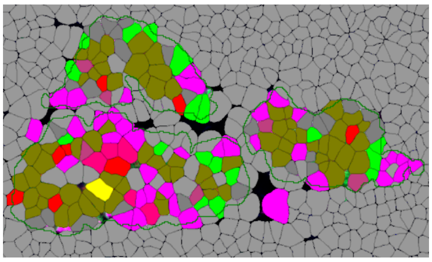

Green: Ins+

Red: Proins+

Magenta: Gluc+

Olive: Ins+ Proins+

7. Export data:

- Press [icon] and select *show annotation measurements*.
- Select *Copy to clipboard* and paste the data on an excel file.

8. Analysis of immune cells by machine learning:

- Create a new class, in our case, CD45+.
- Use the ignore* class for negative cells.
- Go to *Classify* → *Object classification* →*Train object classifier*.
- Select in *Training* the option *Points only*.

- To begin with the training, use the default settings. For more details on the different options, check the QuPath website.
- Press *Live update*.
- Go to the tool bar and select 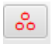.

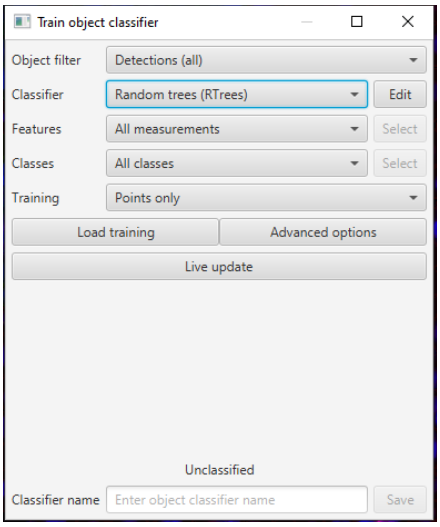

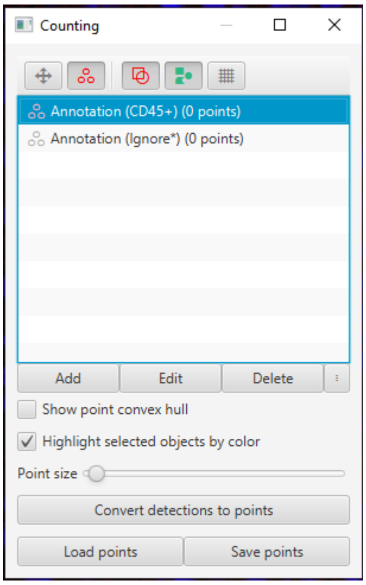

- Start marking positive and negative cells. For the ignore* class it is important to mark beta cells, alpha cells, exocrine cells and all the cells that are not considered positive (CD45+ in our example).
- Once positive and negative cells are correctly detected, save the classifier and apply it.
- Press 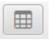 and select show annotation measurements.
- Copy to clipboard and paste the data on an excel file.

9. Spatial analysis of immune infiltration

- First detect the islets (if not performed in previous steps).
- Run positive cell detection for the marker of interest (e.g. CD3). Please, check QuPath's website for details on this option.
- After positive cells are detected, go to *Analyze → Spatial analysis → Distance to annotations 2D*.
- Select *Distance to annotation with islet µm* to see a heatmap showing the color-coded cells based on their distance to the closest islet. * It is important to right

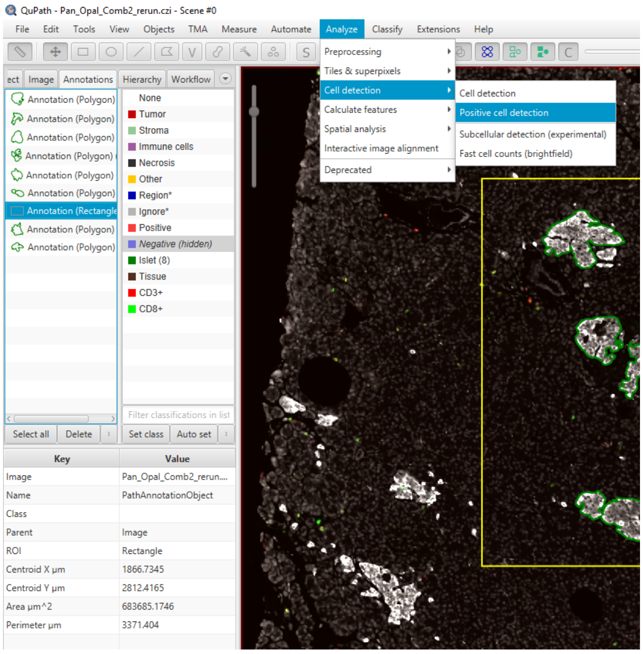

click on the Negative class in the left panel and press hide, in order to make the negative cells hidden.

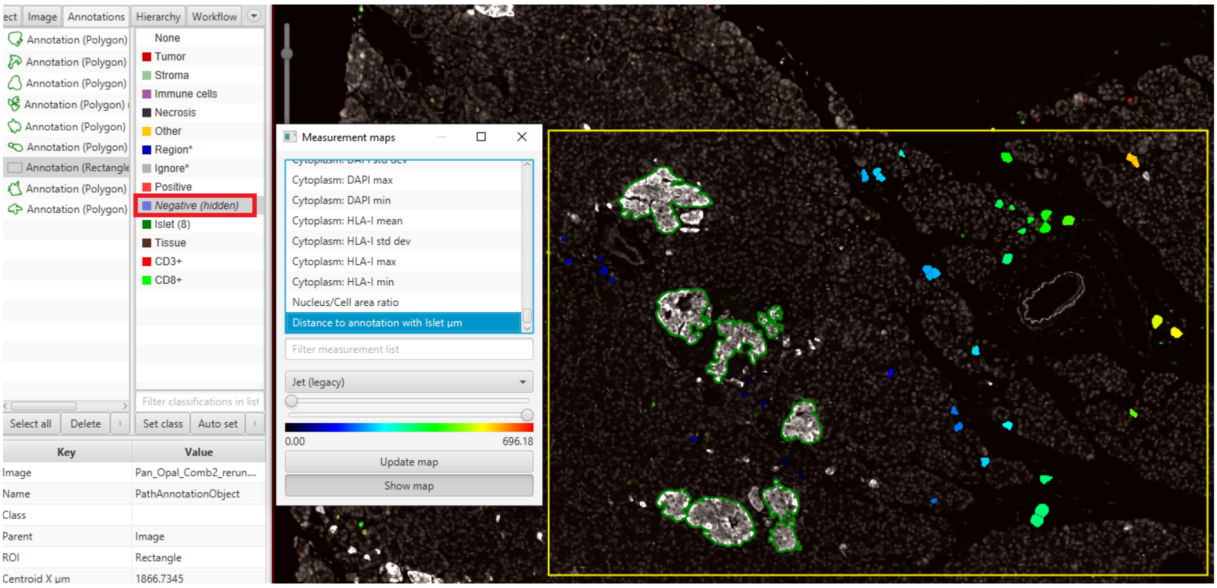

- Export the detection measurements to obtain the distance of every single immune cell to the closest islet.
